# Supplementary material for: COVID-19 Related Myocarditis in Adults: A Systematic Review of Case Reports
Source: J Clin Med. 2022 Sep 21;11(19):5519. doi: 10.3390/jcm11195519 (PMC9573317; doi:10.3390/jcm11195519)
Supplement: Supplementary file 1 [file jcm-11-05519-s001.zip › jcm-1908176-supplementary.pdf]

LP	Title	Author (one surname et al.)	Number of reported cases
1.	Myocarditis in a patient with COVID-19: a cause of raised troponin and ECG changes.	Doyen et al.	1
2.	Diffuse myocardial inflammation in COVID-19 associated myocarditis detected by multiparametric cardiac magnetic resonance imaging.	Luetkens et al.	1
3.	COVID- 19-related myocarditis in a 21-year-old female patient.	In-Cheol et al.	1
4.	Acute myocarditis presenting as a reverse Tako-Tsubo syndrome in a patient with SARS-CoV-2 respiratory infection.	Sala et al.	1
5.	Endomyocardial biopsy findings in Kawasaki-like disease associated with SARS-CoV-2.	Bonet et al.	1

6.	Coronavirus disease (COVID-19) associated delayed-onset fulminant myocarditis in patient with a history of severe acute respiratory syndrome coronavirus 2 (SARS-CoV-2) infection	Ishikura et al.	1
7.	Presence of active myocarditis at the 6 month follow-up appointment for a severe form of COVID-19: a case report	Meyer et al.	1
8.	COVID-19-related myocarditis post-heart transplantation	Roest et al.	1
9.	Cardiac involvement in a patient with coronavirus disease 2019 (COVID-19)	Inciardi et al.	1
10.	Coronavirus fulminant myocarditis saved with glucocorticoid and human immunoglobulin	Hu et al.	1
11.	First case of COVID-19 complicated with fulminant myocarditis: a case report and insights.	Zeng et al.	1

12.	Clinical and Histopathologic Features of Myocarditis in Multisystem Inflammatory Syndrome (Adult)-Associated COVID-19	Aldeghaither et al.	1 of 3
	Clinical and Histopathologic Features of Myocarditis in Multisystem Inflammatory Syndrome (Adult)-Associated COVID-19	Aldeghaither et al.	2 of 3
	Clinical and Histopathologic Features of Myocarditis in Multisystem Inflammatory Syndrome (Adult)-Associated COVID-19	Aldeghaither et al.	3 of 3
13	Clinically suspected myocarditis in COVID-19 patients: Case series and review of the literature	Amin et al.	1 of 11
	Clinically suspected myocarditis in COVID-19 patients: Case series and review of the literature	Amin et al.	4 of 11

Clinically suspected myocarditis in COVID-19 patients: Case series and review of the literature	Amin et al.	6 of 11
Clinically suspected myocarditis in COVID-19 patients: Case series and review of the literature	Amin et al.	7 of 11
Clinically suspected myocarditis in COVID-19 patients: Case series and review of the literature	Amin et al.	8 of 11
Clinically suspected myocarditis in COVID-19 patients: Case series and review of the literature	Amin et al.	9 of 11
Clinically suspected myocarditis in COVID-19 patients: Case series and review of the literature	Amin et al.	10 of 11

	Clinically suspected myocarditis in COVID-19 patients: Case series and review of the literature	Amin et al.	11 of 11	
14	Case Report: Co-occurrence of Myocarditis and Thrombotic Microangiopathy Limited to the Heart in a COVID-19 Patient	Menter et al.		1
15	Case report: high-grade atrioventricular block in suspected COVID-19 myocarditis.	Ashok et al.		1
16	Management of COVID-19 myopericarditis with reversal of cardiac dysfunction after blunting of cytokine storm: a case report.	Li et al.		1
17	Microthrombosis as a cause of fulminant myocarditis-like presentation with COVID-19 proven by endomyocardial biopsy COVID confirmed by antigen test (not PCR)	Nakatani et al.		1

18	Delayed acute myocarditis with COVID-19 infection	Ismayl et al.	1
19	Fulminant Delta COVID-19 myocarditis: a case report of fatal primary cardiac dysfunction	Thomson et al.	1
20	Arrhythmogenic Cardiomyopathy as a Late Complication of COVID-19-Induced Myocarditis	Campoamor et al.	1
21	Veno-arterial extracorporeal membrane oxygenation for COVID-19-associated acute myocardial injury complicated by refractory cardiogenic shock	Flagiello et al.	1

22	Cardiogenic Shock due to COVID-19-Related Myocarditis in a 19-Year-Old Autistic Patient	Pascariello et al.	1
23	Successful heart transplantation for COVID-19-associated post-infectious fulminant myocarditis	Gaudriot et al.	1

24	Myocarditis and Pericarditis in Patients with COVID-19	Shah et al.	3 (2 included)
25	Myopericarditis and myositis in a patient with COVID-19: a case report	Shabbir et al.	1
26	SARS-Cov-2 fulminant myocarditis: an autopsy and histopathological case study	Gauchotte et al.	1
27	COVID-19 fulminant myocarditis: a case report	Bernal-Torres et al.	1

28	Myocarditis Associated With COVID-19	Oleszak et al.	1
29	Fulminant myocarditis in a patient with coronavirus disease 2019 and rapid myocardial recovery following treatment	Gay et al.	1
30	Cardiogenic Shock Caused by SARS-CoV-2 in a Patient with Serial Negative Nucleic Acid Amplification Tests. Case Report	Gomila-Grange et al.	1

31	An Atypical Presentation of Fulminant Myocarditis Secondary to COVID-19 Infection	Richard et al.	1
32	Evidence of SARS-CoV-2 mRNA in endomyocardial biopsies of patients with clinically suspected myocarditis tested negative for COVID-19 in nasopharyngeal swab	Wenzel et al.	2
33	Case report: lymphohistiocytic myocarditis with severe cardiogenic shock requiring mechanical cardiocirculatory support in multisystem inflammatory syndrome following SARS-CoV-2 infection.	Bemtgen et al.	1

34	Acute myocarditis associated with COVID-19 infection	Beşler et al.	1
35	Focal myocarditis in a young male with SARS-CoV-2 infection.	Meel et al.	1
36	Myocarditis revealing COVID-19 infection in a young patient.	Paul et al.	1
37	A Recovered Case of COVID-19 Myocarditis and ARDS Treated with Corticosteroids, Tocilizumab, and Experimental AT-001.	Coyle et al.	1
38	Fulminant myocarditis due to COVID-19.	Irabien-Ortiz et al.	1

40	Virus-Negative Necrotizing Coronary Vasculitis with Aneurysm Formation in Human SARS-CoV-2 Infection	Frustaci et al.	1
41	Myocarditis detected after COVID-19 recovery.	Sardari et al.	1
42	Terra incognita: clinically suspected myocarditis in a SARS-CoV-2 positive patient.	Warchoł et al.	1
43	Management of a critically ill patient with COVID-19-related fulminant myocarditis: A case report.	Shahrami et al.	1
44	COVID-19-associated myocarditis presenting as new-onset heart failure and atrial fibrillation.	Gaine et al.	1

45	ST elevation revealing acute myocarditis with SARS cov 2 infection: Case report.	Taouihar et al.	1
46	COVID-19 myocarditis and postinfection Bell's palsy.	Dahl et al.	1
47	Delayed Acute Perimyocarditis and Bilateral Facial Nerve Palsy in a Patient with COVID-19.	Matsumura et al.	1
48	Acute Perimyocarditis in a Case of Multisystem Inflammatory Syndrome in Adults.	Praet et al.	1

49	Self-limiting COVID-19-associated Kikuchi-Fujimoto disease with heart involvement: case-based review.	Masiak et al.	1
50	Stemi mimicking post COVID: a case of focal myopericarditis post COVID infection.	Atif Masood Noori et al.	1
51	Suspected COVID-19-Induced Myopericarditis.	Okor I. et al.	1

52	COVID -19 Complicated By Acute Respiratory Distress Syndrome, Myocarditis, and Pulmonary Embolism. a Case Report.	Bulbul et al.	1
53	Coronavirus disease 2019 (Covid-19) presenting as purulent fulminant myopericarditis and cardiac tamponade: A case report and literature review.	Khatri et al.	1
54	Delayed acute myocarditis and COVID-19-related multisystem inflammatory syndrome.	Nicol et al.	1
55	Case report of severe PCR-confirmed COVID-19 myocarditis in a European patient manifesting in mid January 2020.	Hudowenz et al.	1
56	Fatal lymphocytic cardiac damage in coronavirus disease 2019 (COVID-19): autopsy reveals a ferroptosis signature.	Jacobs et al.	1
57	Coronavirus (COVID-19) Fulminant Myopericarditis and Acute Respiratory Distress Syndrome (ARDS) in a Middle-Aged Male Patient.	Hussain et al.	1

58	Atypical Manifestation of COVID-19-Induced Myocarditis.	Rehman et al.	1
59	Subacute perimyocarditis in a young patient with COVID-19 infection.	Monmeneu et al.	1
60	Myocardial localization of coronavirus in COVID-19 cardiogenic shock.	Tavazzi et al.	1

Demographic data	Country	Origin/race/ethnicity	Age (years)	Sex (m/f)
	Italy	NR	69	m
	NR	NR	79	m
	NR	NR	21	f
			43	f
	NR	NR	19	m

NR	NR	45	m
NR	NR	81	m
NR	NR	50	NR
NR	NR	53	f
NR	NR	37	m
NR	NR	63	m

USA	African	39	f
USA	Caucasian	25	m
USA	Caucasian	21	m
Iran	NR	18	f
Iran	NR	32	m

Iran	NR	34	f
Iran	NR	22	m
Iran	NR	45	m
Iran	NR	28	m
Iran	NR	51	f

Iran	NR	39 f
Switzerland	NR	47 f
United Kingdom	NR	53 m
USA	NR	60 m
Japan	NR	49 m

USA	NR	53	m
	Japanese ethnicity	39	f
Portugal	NR	32	m
France	NR	30	f

	Italian	NR	19 m
	France	Democratic Republic of Congo	38 m

Qatar	NR	19/38	m/m
England	Afro-Caribbean	50	f
France	NR	69	m
Colombia	Mestizo	38	f

USA	Afro-American	52 m
USA	NR	56 m
Spain	Uruguay	39 m

USA	Caucasian		28 f
Germany	NR	39/36	m/m
Germany	NR		18 m

Turkiye	NR	20	m
South Africa	Indian	31	m
France	NR	35	m
USA	NR	57	m
Spain	NR	59	f

Italy	NR	50 f
Iran	NR	31 m
Poland	NR	74 m
Iran	NR	78 m
Australia	NR	58 m

Morroco	NR	51	m
Norway	NR	37	m
Japan	Japanese	41	m
Belgium	NR	31	f

Poland	Caucasian	43	m
USA	NR	44	m
USA	NR	72	f

Qatar	NR	49 f
USA	NR	50 m
France	NR	40 m
Germany	NR	48 m
Belgium	NR	48 m
USA	Italian	51 m

	USA	Kenyan	39 m
	NR	NR	43 m
	Italy	NR	69 m

Clinical characteristic	Reported symptoms	Physical examination findings (Temp, HR,RR, SpO2%, auscultation findings etc)
	vomiting and diarrhoea 7 days before, cough, fever of 39°C, dyspnoea,	
	fatigue, shortness of breath, recurrent syncopes,	
	febrile sensation, coughing, sputum, diarrhoea, shortness of breath	
	oppressive chest pain, dyspnoea	
	recurrent fever, cough, cervical adenopathy, chest pain and dyspnoea	

	NR	
	no symptoms	
	acute symptoms of cardiac decompensation	
	severe fatigue, fever and cough week before	
	chest pain and dyspnoea for 3 days, accompanied by diarrhoea	
	expectoration of the white sputum, shortness of breath, chest tightness after activity,	

	fever, dyspnoea, chest pain, diarrhea	
	dyspnoea, fever, hypotension	
	dyspnoea, fever	
	loss of consciousnes, fever, headache	
	syncope after an episode of chest pain with fever and myalgia, dyspnoea, dry cough	

	losing consciousness, fever and headache for six consecutive days	
	dry cough, dyspnoea	
	fever, dyspnoea, pleuritic chest pain	
	chest pain, fever, dry cough	
	fever, cough, cardiopulmonary arrest	

	chest pain, cough, orthopnea	
	oligosymptomatic flu-like disease for a week, then - she was found unconscious and apneic at home	
	fever and right upper quadrant abdominal pain	
	8 days of fever, cough, and worsening dyspnoea, with mild abdominal pain and diarrhoea despite completing a 3-day course of azithromycin as an outpatient. He denied chest pain, nausea, or vomiting.	
	NR	

	chest pain, diaphoresis, intense fatigue, and body aches	
	1-day history of profuse diarrhoea, vomiting, abdominal pain, and no respiratory symptoms	
	acute chest pain, palpitations	
	fever, chest pain, and worsening dyspnoea for 5 days	

	worsening of the flu syndrome, and showed up with fever, cough, accompanied by diarrhea and vomits for 3 days.	
	chest pain and vomiting	

	7-day history of fever, generalized weakness, cough, and shortness of breath/ chest pain, not related to breathing, exertion, or posture	
	4-day history of central chest pain, which was made worse on lying flat and on deep inspiration	
	fever, asthenia, and abdominal pain that had been progressing for a week.	
	palpitations that started suddenly 3 days prior to presentation and were associated with haemodynamic instability	

	cough, subjective fever, shortness of breath, and a single episode of trace hemoptysis over the last 5 days	
	dyspnoea and lethargy	
	fever, right flank pain and diarrhoea of 6 days	tachycardia and hypotension

	she was found to be lethargic and covered in coffee ground emesis at home	Breath sounds were clear to auscultation bilaterally. Heart sounds were tachycardic, with a regular rhythm, and no appreciable murmurs. Her abdomen was nondistended, soft, nontender, with a nonpalpable liver and spleen. The patient had no peripheral edema in the extremities, which were cool to touch with 1+ peripheral pulses bilaterally. There were no focal neurological deficits found on the physical examination.
	shortness of breath	
	hyperpyrexia (42◦C), chills and tachycardia	

	febrile sensation<br>and chest pain	
	exertional dyspnoea	
	chest pain and fatigue	
	shortness of breath, fevers,<br>cough,<br>myalgias, decreased appetite,<br>nausea, and diarrhea<br>for 1 week	
	feverish feeling that had lasted 5 days, accompanied by squeezing anginal chest pain in the absence of respiratory symptoms	

	fever, dry cough and shortness of breath	
	dyspnoea on exertion and low-grade fever	
	hemodynamically unstable new-onset ventricular tachycardia (VT) lasting 12 hours	
	shortness of breath, cough, anosmia, and myalgia	
	recent-onset palpitations and progressive dyspnoea	

	intense epigastric pain at rest and effort, associated with nausea and vomiting progressing for 5 days	bilateral rhonchis
	fever, headache and a unilateral, painful neck swelling	redness in the posterior palate and a tender, non-fluctuant swelling extending from the left mandibular angle towards the sternocleidomastoid muscle
	general fatigue and a high fever	mild edema
	fever, cough, and rapidly progressive shortness of breath. One week earlier, she had experienced an episode of cramping abdominal pain and diarrhea.	tachycardia and bibasal crepitations were noted

	Anosmia and ageusia for 1 day, mild fever for a couple of days. All symptoms disappeared completely within a few days. Five weeks later he was admitted to the hospital due to fever with sweats, sore throat, fatigue, dyspnoea, dry cough, and skin changes for 8 days prior to the hospitalization. Additionally, he reported discomfort in the right lower abdomen, discoloured stools, and dark-colored urine.	
	dry cough associated with generalized body ache but denied any chest pain, shortness of breath, palpitation, orthopnea, or fever	
	worsening shortness of breath,	

	cough, shortness of breath	
	fevers, chills, generalized malaise, non-productive cough, dyspnoea for 3-4 days and an episode of near-syncope	
	fever, odynophagia, and left neck pain	
	high-grade fever, dyspnoea and haemoptysis	
	fever, diarrhoea, cough, dysosmia, and dyspnoea	
	dry cough, fatigue, dyspnoea, and a fever	

	midsternal chest pain	
	fever for 14 days, dry cough, haemoptoic sputum,	
	worsening dyspnoea, persistent cough, weakness since 4 days,	

Temp [°C]	HR [/min]	RR [mmHg]	SPO2[%]	auscultation findings	other
39.0	NR	NR	98.2	NR	NR
35.6	75	101/64	94.0	moderate wheezing	NR
NR	NR	NR	NR	NR	NR
37.7	79	120/80	89.0	decreased breath sounds at lung bases with ronchi	NR
39.0	140	81/43	NR	NR	NR

36.6		147	73/34	NR	NR	ABG pO2 82mmHg, pCO2 32mmHg PaO2/FiO2 ratio 166
NR	NR		NR	NR	NR	NR
NR	NR		NR	NR	NR	NR
36.6		100	90/50	96.0	NR	ABG pH: 7.46, pO2 82mmHg, pCO2 32mmHg, lactate 17.1mg/dL
NR	NR		80/50	NR	NR	CKMB 112.9 ng/L
39.3	NR		NR	91.8	NR	ABG: pH 7.049, PCO2 76,4mmHg, PO2 66,2mmHg; ALT 97 U/L; creatinine 157 umol/L

NR	130	MAP 53	NR	NR	NR
40.5	155	MAP 55	NR	NR	NR
39.8	135	MAP 55	normal	NR	NR
36.8	110	80/60	80.0	rales	GCS 11
36.8	120	85/60	80.0	NR	GCS 13

36.7		114	80/60	82.0	NR	GCS 8
36.8		120	70/50	90.0	NR	NR
38.5		90	100/70	85.0	NR	NR
36.8		92	100/60	92.0	NR	NR
NR	NR		NR	NR	NR	NR

37.2		125	100/60	90.0	NR	NR
NR	NR		NR	NR	NR	NR
NR	NR		NR	NR	NR	NR
afebrile with maximum temperatur e of 37.4		96	NR	87.0	clear lungs and tachypnoea	NR
NR	NR		NR	NR	NR	NR

38.9	132	77/46	90.0	NR	respiratory rate, 26 breaths/min
36.6	120	82/69	98.0	normal heart sounds with no murmurs, no pericardial rub,	NR
36.6	200	77/41	98.0	NR	NR
NR	110	80/50	96.0	NR	NR

40.0	140	110/70	90.0	NR	NR	
NR	130	NR	NR	NR	NR	

38.3	126/ 102	76/44 / 121/93	92.0/ 98.0	not significant (both)	NR
afebrile	85	116/74	97.0	normal	NR
NR	NR	NR	NR	NR	NR
36.5	137	98/54	95.0	oft inspiratory crackles bibasally	NR

36.7	118	161/109	87.0	trace bibasilar rales	NR
NR	NR	NR	66.0	NR	NR
38.6	tachycardia	hypotension	NR	NR	NR

afebrile		144	70/38	90.0 on 15L nonrebreather mask	normal	NR
NR	NR		NR	NR	NR	NR
42.0	NR		NR	NR	NR	NR

39.0		111	146/63	97.0	NR	NR
NR		76	122/76	85.0	NR	NR
NR	NR		NR	NR	NR	NR
NR	NR		NR	NR	NR	NR
NR	NR		75/53	96.0	normal pulmonary ausculation	NR

NR		75	NR	NR	NR	NR
37.8		70	110/80	96.0	NR	NR
NR	NR		NR	NR	NR	NR
37.2		110	170/85	72.0	NR	NR
NR		152	117/97	97.0	bibasal fine crepitations	NR

37.2		19	123/60	70.0	bilateral rhonchis	NR
41.0		115	119/76	100	unremarkable	NR
38.2		132	124/72	NR	NR	NR
NR	tachycardia		NR	NR	NR	NR

39.5	97	100/75	94.0	NR	jaundice in the skin and sclerae, skin erythema on the right side of the neck, chest and back, hepatomegaly (3 cm under the rib arch), without peripheral edema. A palpable enlarged right supraclavicular lymph node—hard, about 2 cm in diameter, and several cervical lymph nodes about 1 cm were noted
36.6	76	139/82	97.0	NR	NR
36.6	132	145/80	98.0 on 15 L O2 (non-rebreather mask)	NR	NR

NR	NR	NR	NR	NR	NR
NR	NR	NR	NR	NR	NR
39.9	123	110/60	NR	NR	tonsillitis with cervical adenopathy
39.0	100	90/65	NR	NR	NR
38.6	NR	NR	87.0	NR	NR
39.6	97	141/89	91.0	bilateral wheezing and rhonchi	NR

36.6	stable	stable	stable	NR	NR
36.8	77	135/80	91.0	NR	NR
NR	NR	NR	NR	NR	NR

Medical history (commorbidities, previous surgeries, events)	Vaccination status (yes/no/not completed)	Type of vaccine	Type of vaccination (producer)
hypertension	no		
asthma	no		
NR	no		
NR	no		
NR	no		

NR	no
hypertension, prostate adenocarcinoma	no
6 years post heart transplantation	no
No previous history of cardiovascular disease	no
NR	no
allergic cough for 5 years, previous smoking history	no

NR	NR	NR	NR
NR	NR	NR	NR
NR	NR	NR	NR
NR	NR	NR	NR
NR	NR	NR	NR

NR	NR	NR	NR
No previous medical history	NR	NR	NR
NR	NR	NR	NR
NR	NR	NR	NR
NR	NR	NR	NR

asthma	NR	NR	NR
obesity [body mass index (BMI) 31.6], depression	NR	NR	NR
No previous medical hi	NR	-	-
hypertension and hyperlipidaemia	NR	-	-
No previous medical hi	first dose of COVID-19 vaccination (9 days before)	NR	NR

No previous medical hi	NR	NR	NR
laparotomy with ovarian cystectomy	no		
After covid-19 exhibited only dry cough and odynophagia, which resolved after symptomatic treatment with paracetamol. otherwise healthy	NR	NR	NR
No previous medical hi	NR	NR	NR

autism spectrum	no
suggested chronic lymphopenia	no

No previous medical hi	no
hypertension, previous myocarditis, reactive arthitis	no
diabetes, hypertension, ischemic heart disease	no
No previous medical hi	no

hypertension, previous myocarditis, reactive arthitis	no
obesity, hyperlipidemia	no
former smoker, 68 g daily alcohol use	no

diabetes mellitus type 1, diabetic gastroparesis, asthma, anxiety, depression with multiple previous episodes of DKA, questionable history of IV drug use	no
Both patients were obese and had a history of upper airway infection with headache, fever, and cough up to 4 weeks before admission.A; dyslipidemia B: hypertension, smoking dyslipidemia, CAD heart failure, lung disease	no
NR	no

NR	no
NR	no
NR	no
hypertension	no
hypertension, cervical degenerative arthropathy, chronic lumbar radiculopathy, lymph node tuberculosis diagnosed due to erythema nodosum, and migraine	no

no	no
NR	no
atrial fibrillation, catheter ablation performed 3 times, arterial hypertension, type 2 diabetes, and hypothyroidism	no
hypertension	no
No previous medical hi	no

hyperthyroidism	NR	NR	NR
No previous medical hi	NR		
hypertension	no		
No previous medical hi	NR		

No previous medical hi	NR		
NSTEMI 2 weeks before, COVID pneumonia 1 month ago	NR		
hypertension, chronic obstructive pulmonary disease	NR	NR	NR

NR	NR	NR	NR
hypertension and ischemic stroke.	NR	NR	NR
obesity (BMI 34.8)	NR	NR	NR
NR	no		
hypertension	no		
hypertension	no		

NR	no
NR	no
NR	no

Diagnostics	Covid-19 confirmation technique (PCR, serology etc.)	myocarditis during COVID (yes/no)
	PCR	yes
	PCR	yes
	PCR	yes
	PCR	yes
	PCR	no

PCR	no
NR	no
PCR	no
PCR	yes
PCR	yes
PCR	yes

NR	no
NR	no
PCR	yes
PCR	yes
PCR	yes

	PCR	yes
	PCR	yes
	PCR	yes
	PCR	yes
	PCR	yes

PCR	yes
PCR	yes
PCR	yes
PCR	yes
antigen test on the nasopharyngeal swab	yes

	PCR	no
	PCR	yes
	NR	no
	PCR	yes

	PCR	yes
	serology	no

	Nasopharyngeal swab/ COVID prior to myocarditis	yes/no
	PCR	no
	COVID found in biopsy	no
	PCR	yes

	PCR	yes
	rapid antigen test	yes
	serology	yes

	COVID in history	no
	PCR negative, COVID found in biopsy	no
	anti-S1 and anti-N antibodies	no

	Nasopharyngeal swab	yes
	NR	no
	PCR	yes
	tested positive, type of test unknown	yes
	PCR	yes

	PCR	yes/no
	PCR	no
	PCR	yes
	nasopharyngeal swab	yes
	PCR	yes

	PCR	yes
	PCR	yes
	PCR	no
	serology	no

	COVID in history	no
	COVID in history	no
	COVID in history	no

	PCR	yes
	PCR	yes
	PCR negativ on admission, IgG positive	no
	PCR testing for SARS-CoV-2 RNA was initiated on the histological specimens of the heart.	yes
	PCR	yes
	PCR	yes

	PCR	yes
	PCR	yes
	PCR	yes

myocarditis post covid (how long)	Inflamatory markers (CRP, WBC, IL-6, Prokalcitonin)	Cardiac markers (troponin, NT-proBNP, Ddimer)	troponin	NT-proBNP
	14·9 × 10^9 per L		9002ng/L (normal < 40)	
	CRP 13.8 mg/L -> 64.23mg/L, WBC - normal ->14.6g/L		18.8 ng/L ->63,5ng/L	normal -> 1178pg/mL
	NR		1.26 ng/mL (normal <0.3ng/mL)	1929 pg/mL (normal <125 pg/mL)
	CRP 18.6 mg/L		135-107-106 ng/L (normal < 14)	512 pg/mL (normal < 153)
4 weeks	ferritin 4560ug/L (normal < 300), procalcitonin 155 ug/L (normal <0.5), type 1 interferon 6.1 (normal <1.7), neutrophil count 24 x 10^9/L (normal <8), lymphopenia 0.5 x 10^9 (normal >1)		4200 ng/L (normal <79)	17377 (normal <500)

5 weeks	WBC 18.600/uL (neutrophils 94.8%), IL-6 533.3 pg/mL (normal <= 4.0 pg/mL), CRP 57.5 mg/dL (elevated)		31.516pg/ml (normal <= 30pg/mL)	NR
6 months	NR		NR	NR
5,5 months	NR		55 ng/l	212 pmol/l (elevated)
	CRP 1.3 mg/dL (Reference range <0.5 mg/dL)		0.24ng/ml (Reference range <0.01ng/ml)	5647 pg/mL (Reference range <300pg/mL)
	NR		> 10000 ng/L (elevated)	21025 ng/L (elevated)
	NR		11.37 g/L (elevated)	22.600 pg/mL (elevated)

4 weeks (asymptomatic)	CRP 283mg/L, ferritin 630ug/L		11810ng/L	NR
5 weeks (asymptomatic)	CRP 315mg/L		1557ng/L	NR
	CRP 185mg/L, ferritin 824ug/L		27000ng/L	NR
	WBC 18000/µl (Reference Range 4500–11,000), CRP 50 mg/L (Reference Range 0.8– 1mg/L), PCT 2 ng/ml (Reference Range < .05), IL-6 24pg/ml (Reference Range < 6)		3xUNL	21.000 pg/ml (Reference Range <125 pg/ml)
	WBC 12400/µl (Reference Range 4500–11,000), CRP 65 mg/L. (Reference Range 0.8– 1mg/L), PCT 0,2 ng/ml (Reference Range < .05), IL-6 24 pg/ml (Reference Range < 6)		2xUNL	10.000 pg/ml (Reference Range <125 pg/ml)

	WBC 1300/µl (Reference Range 4500–11,000), CRP 45 mg/L (Reference Range 0.8– 1mg/L), PCT 0,2 ng/ml (Reference Range < .05), IL-6 20 pg/ml (Reference Range < 6)		4xUNL	3.000 pg/ml (Reference Range <125 pg/ml)
	WBC 10700/µl (Reference Range 4500–11,000), CRP 55mg/L (Reference Range 0.8– 1mg/L), PCT 2 ng/ml (Reference Range < .05), IL-6 24 pg/ml (Reference Range < 6)		3xUNL	21.000 pg/ml (Reference Range <125 pg/ml)
	WBC 11000/µl (Reference Range 4500–11,000), CRP 30 mg/L (Reference Range 0.8– 1mg/L), PCT 0,8 ng/ml (Reference Range < .05), IL-6 12 pg/ml (Reference Range < 6)		2xUNL	4.300 pg/ml (Reference Range <125 pg/ml)
	WBC 2400/µl (Reference Range 4500–11,000), CRP 3.5 mg/L (Reference Range 0.8– 1mg/L), PCT 0,2 ng/ml (Reference Range < .05), IL-6 10 pg/ml (Reference Range < 6)		2xUNL	1.200 pg/ml (Reference Range <125 pg/ml)
	WBC 1800/µl (Reference Range 4500–11,000), CRP 70 mg/L (Reference Range 0.8– 1mg/L), PCT 2,2 ng/ml (Reference Range < .05), IL-6 24 pg/ml (Reference Range < 6)		3xUNL	22.000 pg/ml (Reference Range <125 pg/ml)

	WBC 12000/µl (Reference Range 4500–11,000), CRP 103mg/L (Reference Range 0.8– 1mg/L), PCT <0,1 ng/ml (Reference Range < .05), IL-6 8 pg/ml (Reference Range < 6)		2xUNL	23.000 pg/ml (Reference Range <125 pg/ml)
	CRP 282 mg/l (elevated)		507 ng/l (elevated)	>70,000 ng/l (elevated)
	CRP 529 mg/L elevated, WBC 15.4 (elevated),		2.6 ng/L (normal)	NR
	WBC (x10^3) 17.9 (3.8–10.5), CRPmg/dL 29.35 (<0.40), Ferritin ng/mL 6379 (15–150),		582 ng/L (<6 – 14)	15642pg/mL (0-300)
	CRP 1.685 mg/dL (normal range ≤0.180 mg/dL), WBC 7100 (normal range 4000-8000/µL)		1.010 ng/mL, norm: ≤0.014 ng/mL	NT-proBNP 27541.0 pg/mL, norm: ≤125.0 pg/mL

35 days	WBC 25.1 k/lL, CRP >375 mg/L		2.83 ng/mL (normal <0.04 ng/mL), with a peak of 13.6 ng/mL	60,230 pg/mL
	CRP 1.6		118 ng/L (norm: <14 ng/L	6,543 (norm: <125)
40 days	NR		149.87 (norm: 24 - 30 pg/mL)	70.89 (norm: < 125 pg/mL)
	WBC 14.9 (elevated)		5891 ng/L (elevated)	16569 ng/L (elevated)

	WBC, 15.59 × 103/µL (4.0 - 10.8) , CRP 401 mg/L (0.7 - 1.2)		1033 ng/ml (< 14 ng/L)	47650 ng/ml (< 300)
5 weeks	CRP 60 mg/L (elevated), ferritin 6200 µg/L		1600 pg/ml (<14 pg/mL)	10500 pg/ml (<300 pg/mL)

no/3 weeks	1. WBC 19.4 (4.0-10.0×103/uL) , CRP 340 (<5 mg/L), Procalcitonin >100 (<0.5 ng/mL) , IL-6 678 (<7 pg/mL), Ferritin 11719 (38-270 ug/L)/ 2. WBC 15,2 (elevated), CRP 3mg/L (N <5)		1. 256 ng/ml (<15 ng/L)/ 2. 1264 ng/L (normal <15)	NR
	WBC 4.98 (4–11 (10^9 cells/L), CRP 58mg/L (0–4), Ferritin 172mg/L (15–300),		77 ng/L (0-14)	NR
no	WBC 14 460/mm3 (elevated); C-reactive protein, 329 mg/L (elevated)		8066 pg/mL (elevated)	NR
	WBC 19000x 10^3/uL (4.5–11.3 x 10^3/uL), CRP 25.7 (0–5 mg/mL)		1190 ng/ml (<14 ng/L)	BNP - 13000 pg/ml (<100 pg/mL)

	CRP 104.97 mg/L (elevated), procalcitonin 1.95 ng/ml (elevated), ferritin 1492 ng/ml (elevated)		0.017 ng/ml (elevated)	proBNP 1220 pg/ml (elevated)
	WBC 9.8 K/µL, CRp 4.1 procalcitonin 1.4 - increased		troponin I 1.3 ng/mL - inscreased	BNP 790 pg/ml - increased
	leucocytosis of 22.9 × 109/L (neutrophils 92%, lymphocytes 3.1%) ferritin (3188 ng/mL, N < 400), CRP 33 mg/dl		troponin I raised to 3800 ng/mL [N < 13 ng/mL]	(pro-BNP) to 27,696 pg/mL (N < 125)

yes (time not reported)	WBC 29 x 103/uL (reference range: 4.5-11 x 103/uL), CRP2.47 mg/dL (reference range: 0.0-0.74 mg/dL),ferritin of 119 ng/mL (reference range: 11-307 ng/mL)		0.04 ng/mL (reference range: <0.04 ng/mL)	NR
aprox 30 days	WBC 13/8.61 ×10*9/L (3.5 ×109/L – 10 ×109/L) CRP 1370/200 mg/L (< 50mg/L),	elevated serum levels of natriuretic peptides and cardiac troponin I	0.372/ 0.057 ng/mL (< 0.024ng/mL)	BNP - 109/258 ng/L, (< 100ng/L)
2 months	CRP; 105.9 mg/l, reference range <5 mg/l<br>IL-6; 128 pg/ml, reference range <7 pg/ml<br>PCT; 0.12 ng/ml, reference range < 0.05 ng/ml		341 ng/L, ref <14	NR

	CRP 0.0812 g/L (0.005 g/L) WBC 6.74 × 10^9 per L		0.572 ng/mL (<0.045 ng/mL)	(NT-proBNP) level was 127 ng/L (<125 ng/L)
3 weeks	WBC4.80x 109/l (normal 3.92–10.4) CRP 5 mg/l (normal < 10 mg/l) ferritin 267 µg/l (normal 30–400 ug/l)		319 ng/l (normal < 14 ng/l)	NT-proBNP of 143
no	NR		2885 ng/L (increased)	NR
no	WBC 4.7 (×109/l)4.2–11) CRP 8.1(mg/dl)<1.0) PCT 0.18 (ng/ml) <0.10Ferritin (ng/ml)2 106 (26-388)Interleukin-6 (pg/ml) 18 (0-5)		0.02–7.33 (ng/mL) (<0.05)	859 (pg/ml) (<126)
no	WBC 14.17 × 10^9/L elevated, CRP 10 mg/L elevated,		T 220-1100 ng/dL (elevated)	4421 ng/L (elevated)

no	1.7 mg/dL, nv 0.00–0.50 mg/dL		0.077 mcg/L, nv < 0.014 mcg/L	NR
3 weeks	WBC normal, CRP 3.3 mg/L - normal		T <0.03 ng/mL - normal	NR
no	neutrophil-to-lymphocyte ratio higher than 3 CRP 1.2 mg/l (reference range <6 mg/l) PCT normal		troponin T 72 ng/l (reference range <14 ng/l)	2451 ng/l (reference range <125 ng/l)
no	CRP 163 (<6) mg/L		103 (0–0.3) ng/ml	NR
no	CRP 7 mg/L (normal range: 0–5 mg/L)		T 25 ng/L (normal range: 0–14 ng/L)	3428 pg/mL (normal range: 0–400 pg/mL)

	white lymphopenia at 850/mm3 blood cells at 14.000/µl, C-reactive protein at 120 mg/l (normal between 0.00 and 5.00 mg/, ferritin at 500 µg/		20000 ng/L (normal < 20 ng/L)	NR
	CRP 230 mg/L (0-5mg/L), procalcitonin 2.1 µg/L (0–0.10 µg/L), WBC 7.1x10^9/L (3.5–11.0×10^9 /L)		TnT 90 ng/L (0–15 ng/L)	160 ng/L (0–85 ng/L)
	CRP 24.74 mg/dL (elevated), WBC 14,100 /µL		0.094 ng/mL	NTproBNP 4,938 pg/mL (elevated)
2 months before	CRP 381 381 mg/dL (elevated), ESR >120 mm/h (elevated),		hs-TnT 151 ng/L (elevated)	25,386 pg/mL (elevated)

5 weeks later	leukocytosis with lymphopenia (WBC 14 G/l; lymphocytes 0.6 G/l), (CRP 281 mg/l - elevated, procalcitonin 0.9 ng/ml- elevated, ESR 62 mm/h), ferritin (3400 ng/ml)- elevated		0.119 ng/ml (normal < 0.03 ng/ml)	(BNP) was 965 pg/ml (< 73 pg/ml)
1 month	ESR 25, CRP 3.2 - elevated		11,67 - elevated	NR
1 week	CRP 35.1 mg/L (reference range, 0-8.2 mg/L), ferritin of 394 ng/mL (reference range, 20-300 ng/mL)		1.0 ng/ml	2,336 pg/mL(reference range, 0-99 pg/mL)

-	CRP 193 mg/L (0-5 mg/L), WBC 7.8 (4-103 u/L), PCT 1.38 ng/ml (<0.5 ng/mL)		9 ng/L (3-10 ng/L)	430pg/mL (<125 pg/mL)
-	CRP 11.85 mg/L, WBC 19.290 /µL - elevated, PCT 8.16 ng/ml, ferritin 66,165 ng/ml, OB 46 - all elevated		544 ng/L	NR
4 weeks	CRP 604 mg/L, WBC 25, fibrinogen 12.5 g/L, PCT 14 µg/L, IL-6 75.6 µg/L (N < 7) (all elevated)		485 ng/L (N < 34)	2960 ng/L (elevated)
no	CRP 13.0 mg/dL (0–0.5 mg/dL) Ferritin 468 ng/mL(30–400 ng/mL), Leucocytes 13.8/nL (3.5–10.0/nL)		3264 pg/ml (0–14 pg/mL)	12232 pg/mL (1–300 pg/mL)
no	CRP (mg/L) 247.1 (<5.0) WBC (×10 3 /µL) 10.6 (3.9–10.6) Ferritin 1200µg/L (<322)		14 932 ng/L (< 45 ng/L)	9223 pg/mL (< 125 pg/mL)
no	NR		0.29 ng/mL (elevated)	1,287 pg/mL (elevated)

	CRP 3.3 mg/dL (elevated)		5.97 ng/mL (elevated)	379 pg/mL
	CRP 14mg/L (<10mg/L), leukocyte count 4.03 x 10^3/uL, neutrophils 83% (<70%), lymphocytes 0.42 x 10^3/uL 10.4%		troponin T curve 29-24-24-18-15 ng/L (normal < 14ng/L)	456 pg/mL (<125pg/mL)
	WBC 23080 /mm3, neutrophils 91.4%, lymphocytes 1.4%, CRP 52,7 mg/L		4332 ng/L (elevated)	NR

D-dimer	Other findings/markers	Chest X-ray findings	ECG
			LVH, diffuse inverted T waves
		normal	normal
		multifocal consolidation on both lung fields and cardiomegaly	non-specific intraventricular conduction delay, multiple premature ventricular complexes
		subtle bilateral opacities suggesting interstitial inflammatory lung disease	low atrial ectopic rhythm, mild ST-segment elevation in leads V1-V2 and aVR, reciprocal ST depression in V4-V6, QTc 452ms with diffuse U-waves
NR			

NR	BNP: 3905 pg/mL (normal <= 18.4pg/mL), CK: 2254 U/L (normal 59-248), PCR SARS-Cov-2 negative, SARS-CoV-2 spike and nucleocapsid protein transcripts detected by RT-PCR (myocardial biopsy)		sinus tachycardia with a ventricular rate of 160, ST elevation in any lead
NR	restrictive respiratory d	NR	NR
NR	PCR on plasma and heart biopsy tested negative for SARS-CoV-2 RNA by RT-PCR	NR	NR
	CK-MB 20.3 ng/mL (Reference range <4.9 ng/mL)		low voltage in the limb leads, minimal diffuse ST-segment elevation, ST-segment depression and T-wave inversion in lead V1 and aVR
NR	NR	significant enlargement of the heart	III, aVF ST-segment elevation
			sinus tachycardia and no ST-segment elevation

>4mcg/mL		normal	diffuse ST-segment elevation
>4mcg/mL		normal	sinus tachycardia
3.5mcg/ML		NR	sinus tachycardia
1200 ng/ml (Reference Range <250)	-	NR	sinus tachycardia, low voltage QRS
2400ng/ml (Reference Range <250)			sinus tachycardia, low voltage limb leads, inverted T waves in two limb leads (L1 and aVL), and ST-segment depression in three pericardial leads (V1–V3)

1500ng/ml (Reference Range <250)	-	NR	ventricular tachycardia - 150/min
3500ng/ml (Reference Range <250)	-	NR	sinus tachycardia
1400ng/ml (Reference Range <250)	-	NR	sinus tachycardia, no significant changes in the ST-segment, low-voltage waves in the limb leads, and QRS alternans
1700ng/ml (Reference Range <250)	-	NR	sinus rhythm with ST-segment elevation and Q waves in precordial leads
2400ng/ml (Reference Range <250)	-	NR	torsade de pointes

1350ng/ml (Reference Range <250)	-	NR	sinus tachycardia
NR	thrombocytopenia 140 G/L, aPTT 39 s		ST-segment depression in all the leads and elevations in aVR
NR	-	left upper lobe opacification	on day 7 - type 2 AV block (Mobitz type II), then complete heart block (Type 3) with junctional escape rhythm at 35/min
458 ng/mL (<230)	-	severe bilateral opacities	diffuse new ST-segment elevation, with QTc interval measured at 437 ms
3.8 µg/mL, normal range <1.0 µg/mL	NR	NR	NR

-	-	unremarkable	sinus tachycardia (133/min) with ST elevation in V1 to V3
>10 (norm: <0.25)	Digital subtraction angiography (DSA) of the left leg demonstrated severe vasospasm of the anterior and posterior tibial arteries but patent flow into the foot requiring four-compartment fasciotomies.	normal	normal sinus rhythm (102/min) without ST changes
200 (norm: 0 - 230 ng/m)	NR	normal	ventricular tachycardia - 200/min
NR	NR	NR	sinus tachycardia at 110 and a diffuse ST-segment elevation (more marked in the lateral and inferior leads)

NR	NR	Bilateral interstitial pneumonia with thickening and interstitial infiltrates suggestive of viral pneumonia	NR
	serum creatinine 130 µmol/L, a 10-fold increase in serum transaminases level		an inferior and lateral QS aspect, with an incomplete right bundle branch block

1. 16.44mg/L (<0.44 mg/L)/ 2. NR	AKI creatinine 344/ NR	bilateral congestive changes and infiltrates in the left lower zone/ bilateral infiltrates which were less prominent compared to his previous X-rays when he was admitted with COVID-19 pneumonia	sinus tachycardia at a rate of 150 /min sinus rhythm with diffuse concave ST-segment elevation in multiple leads. PR depression was also noted in inferolateral leads and PR elevation in aVR
NR	CK 6432 U/L (normal value 25–200 U/L	normal	poor R-wave progression anteriorly and small QRS complexes
NR	Arterial blood gas analysis showed compensated metabolic acidosis with hyperlactacidemia (pH, 7.51; excess basis, 9 mEq/L; lactates, 3 mmol/L) and normoxemia. CK-MB 2103 UI/L (elevated)	NR	NR
NR	NR	reticular interstitial opacities	diffuse and concave elevation of the ST-segment, with PR segment depression and Spodick's sign

313 ng/ml (elevated)	Arterial blood gas analysis showed a pH of 7.49, carbon dioxide partial pressure of 27.9 mm Hg, oxygen partial pressure 93.3 mm Hg, bicarbonate level of 24.4 mm Hg, and a lactate level of 1.8 mmol/L.	bilateral multifocal opacities	sinus tachycardia, left ventricular hypertrophy, biatrial enlargement, and QTc 449ms
1961 ng/ml - increased	creatinine 2.0 mg/dL - increased	multifocal bilateral airspace disease	rapid atrial fibrillation and low voltage
(2132 ng/mL, N < 580)	lactate dehydrogenase (552 U/L, N < 225), transaminases (ALT 103 U/I [N < 41], AST 88 U/I [N < 38]), prothrombin time (1.33, [N < 1.2])	normal	normal

NR	creatinine of 4.4 mg/dL (reference range: 0.44-1.00 mg/dL), glucoe 1679 mg/dl, anion gap of 46 mmol/L (reference range: 5-13 mmol/L), carbon dioxide of 6 mmol/L (reference range: 24-31 mmol/L) , potassium of 2.9 mmol/L (reference range: 3.5-5.2 mmol/L), lactic acid level of 17.1 mg/dL, lactate dehydrogenase (LDH) of 296 U/L	findings were suspicious for COVID-19	wide complex tachycardia with a heart rate of 119/min and accelerated junctional rhythm, significant for ST-segment depression in the lateral leads and ST elevation in leads I and aVL, showing sinus tachycardia at 119/min with low voltage and nonspecific ST changes in the lateral leads, and possible septal infarct
NR		NR	T-wave inversions in the anterolateral leads
NR	Pulse Contour Cardiac Output measurement confirmed marginal cardiac output of 4.4 l/min	NR	sinus tachycardia

NR	NR	focal consolidation on the upper zone of left lung	NR
< 0.10 mg/l (0.0–	NR	NR	T-wave inversion in lead III
NR	NR	NR	NR
NR	NR	bibasilar patchy interstitial opacities.	sinus tachycardia without ST-T wave changes
23 242 ng/mL elevated	NR	mild signs of vascular redistribution, with no infiltrations	concave ST-segment elevation and PR-segment depression, as well as low voltages

NR	NR	NR	diffuse and non-specific repolarization abnormalities, QT interval was within limits (460 ms). Holter monitoring showed frequent ectopic ventricular beats and several sequences of non-sustained ventricular tachycardia
NR	NR	NR	NR
1.39 mg/l (reference range <0.5 mg/l)	NR	NR	NR
NR	NR	NR	AF
NR	NR	cardiomegaly, increased interstitial lung markings and small effusions	AF

0.40	NR	NR	ST segment elevation in V1 and V2 derivations with Q waves of necrosis, an abrasion of R waves in V3 and a negative T wave in inferiorterritory.
NR	respiratory alkalosis (on room air: pH 7.55; partial pressure of carbon dioxide 3.9 kPa; partial pressure of oxygen 8.4 kPa; bicarbonate 23 mmol/L).	bibasal consolidations	moderately flattened T-waves
1.1 µg/mL	The main laboratory findings included leukocytosis, liver dysfunction, high levels of N-terminal pro-b-type natriuretic peptide (NT-proBNP), high C-reactive protein and electrolyte abnormality, and dyslipidemia	y showed mild cardiomegaly	sinus tachycardia and slight STsegment elevation in leads I, II, aVL, and V2-6
NR	neutrophilia, lymphopenia, thrombocytopenia, multiplex cytokine panel showed very high CXCL9 and CXCL10 levels as well as signs of inflammasome activation	NR	sinus tachycardia

4613.46 µg/L - elevated	elevated GGTP (386 U/l), alkaline phosphatase (216 U/l) and bilirubin (7.5 mg/dl) with normal transaminase and lactate dehydrogenase levels.	NR	NR
514 - elevated	NR	unremarkable	concave shaped ST-elevation in inferior and lateral leads
0.51 mg/L (reference range, <0.5 mg/L FEU)	NR	NR	normal sinus rhythm shows T wave inversions in inferior and anterolateral leads with prolonged QTc, diffuse ST-elevations

NR	cardiac output at 2.5 L/min measured by pulse contour cardiac output PiCCO, central venous pressure of 24 mmHg, central venous saturation of 50%	bilat- eral infiltrates consistent with COVID-19	wave inversion in the lateral leads
1068 ng/mL	creatine kinase 2135 U/L, creatine kinase myocardial band 54.3 ng/mL	diffuse bilateral patchy opacities	sinus tachycardia, ST-elevation in leads II, III, aVF and ST-depression in I, aVL
5700 ng/L (elevated)	NR	NR	sinus tachycardia
NR	NR	NR	NR
611 µg/L (0–549)	NR	NR	marked QRS widening and a positive deflection at the end of the T wave in the absence of hypokalaemia
NR	NR	bilateral pleural effusion	diffuse ST elevation

0.96 mcg/mL	ESR 44mm/hr, LDH 926u/L, procalcitonin 0,04ng/mL (negative), AST 89 u/L and ALT 152 u/L both elevated, CPK 366 u/L, ferritin 243 ng/mL (normal).	NR	1 to 2mm ST elevations in lead I and aVL, ST depression in aVR, mild J-point elevation, and T-wave inversion in leads II, III and aVF
790ng/mL (<500ng/mL)	NR	interstitial pattern with opacities in both lungs	sinus rhythm with minimal ST-segment elevation and extensive repolarization abnormalities
NR	ABG pH: 7.2, excess basis -6mEq/L, lactates 9mmol/dL and hypoxemia	NR	NR

CT findings	ECHO findings	PCI
bilateral ground-glass opacities and condensations	mild LVH	no disease
pulmonary ground-glass peripheral	normal	
a multifocal consolidation and ground-	severe LV systolic dysfunction	
bilateral patchy ground-glass opacities	mild LV systolic dysfunction (LVEF 43%), with inferolateral wall hypokinesis	
NR	LVEF 20%, low cardiac output	normal coronary arteries

no signs of SARS-CoV-2 infection, atelectasis	severe hypokinesis, LVEF 7.4%	normal coronary arteries
NR	LVEF 39%, global mild hypokinesia and severe inferior wall hypokinesia	NR
NR	biventricular failure and congestion	
NR	LVEF 40%, increased wall thickness, the diffuse echo-bright appearance of the myocardium, diffuse hypokinesis, LV diastolic function mildly impaired with mitral inflow patterns, E/A ratio of 0.7 and E/e' ratio of 12, pericardial effusion (max 11mm) without signs of tamponade,	no evidence of obstructiv e coronary artery disease
pulmonary infection, enlarged heart and pleural effusion	LVEF 27%, enlarged heart LVED 58mm, LAVD 39mm, RVED 25mm, RAED 48mm, trace (2mm) pericardial effusion	NR
NR	LVEF 32%, diffuse myocardial dyskinesia, enlarged left ventricle, pulmonary hypertension 44mmHg	

NR	large pericardial effusion with severe biventricular dysfunction and left ventricular ejection fraction (LVEF) of 10-15% (cardiac tamponade with concurrent cardiogenic shock)	normal coronary arteries
NR	severe LV systolic dysfunction with LVEF 15-20% and moderate RV systolic dysfunction	NR
NR	severe biventricular systolic dysfunction with LVEF 5-10%	NR
diffuse bilateral ground-glass opacities and basal consolidations	small LV with an end-diastolic dimension of 42 mm, LVEF 10% with global hypokinesis, a normal right RV, and pulmonary arterial pressure (PAP), no pericardial effusion (PE) or thrombosis, and no significant valve lesions or dysfunction	NR
diffuse bilateral ground-glass opacities suggestive of COVID-19	LVEF 25%, an enlarged end-diastolic diameter of the left ventricle, normal RV size (mild dysfunction), and normal valvular and pericardial function. Moreover, echocardiography demonstrated increased wall thickness.	NR

bilateral ground-glass opacities and ba	LVEF 30%, normal LV and RV size, moderate tricuspid and mitral regurgitation, systolic pulmonary artery pressure of 30 mmHg	NR
pulmonary edema	LVEF 30%–35%, LVEDD of 5 cm, moderate RV enlargement and dysfunction, dilated inferior vena cava, and mild left-sided pleural effusion	NR
bilateral ground-glass opacities suggestive of viral pneumonia	LVEF of 45%, normal LV size and function without regional wall motion abnormalities, and circumferential pericardial effusion without significant respiratory changes in the mitral and tricuspid valves	NR
bilateral ground-glass opacities	LVEF 30%–35%, hypokinesis in the anterior wall, normal LV size as well as normal RV size and function	normal coronary arteries
bilateral ground-glass opacities	LVEF 35%, normal LV size, increased LV wall thickness	a non-significant (<50%) lesion in the middle part of the left anterior descending artery

bilateral ground-glass opacities	LVEF 30%–35%, mild pericardial effusion, RV enlargement, hypermobile thrombus in the LV (1.5 × 1.5 cm)	normal coronary arteries
excluded pulmonary thromboembolism but showed bilateral lower-lobe consolidations	moderately reduced left ventricular function LVEF 30% and normal right ventricular size and function. There was no evidence of left ventricular hypertrophy or dilatation.	non relevant coronary stenosis
features of acute calculous cholecystitis	LV systolic impairment with no obvious regional wall motion abnormalities	NR
NR	significant for severe segmental LV systolic dysfunction, LVEF 15–20% with hypokinesis of the apex, distal anterior septum, and anterior and lateral walls, with a small pericardial effusion. Right ventricular size and function were normal	NR
bilateral mild ground glass appearance, bilateral pleural effusion and pericardial effusion	low LVEF<20% and diffuse myocardial hypertrophy	no obstructive coronary artery disease

NR	severe global hypokinesis of the left ventricle with LVEF of 25%	Emergent coronary angiogram revealed normal coronary arteries.
CT of the chest reported bilateral patchy consolidation and ground glass opacification in the lower lobes. There were no significant findings on CT of the abdomen and brain.	A severely reduced biventricular function, normal cardiac chamber size, and a globally thickened, bright myocardium (measuring 16 mm in the septum and inferolateral wall) with a small-to-moderate-sized pericardial effusion.	-
NR	dilated LV, mild diffuse hypokinesia with light impairment of global systolic function, and a dilated left atrium	PCI showed no epicardial coronary lesions.
NR	20-mm pericardial effusion compressing the right heart chambers and LV systolic dysfunction LVEF 35% with global hypokinesis	no evidence of significant lesions

The chest computed tomography (CT) ruled out acute pulmonary embolism and revealed cardiomegaly.	enlarged LV (56 mm), diffuse myocardial hypo-akinesia along with severe left ventricular systolic dysfunction LVEF 15-20% and a decrease in right cardiac function	NR
heterogeneous enhancement and thickening of the myocardium, pericardial effusion, bilateral ground glass opacities, posterior consolidations, septal lines, and a moderate right pleural effusion. No evidence of pulmonary embolism was found.	biventricular infiltrative myocardial hypertrophy, associated with a circumferential non-compressive pericardial effusion and a restrictive mitral inflow profile. There was no systolic dysfunction.	not performe d

NR	normal left ventricle size with severe global hypokinesia and severely reduced LV systolic function LVEF of 24%, and there was mild-to-moderate mitral regurgitation. RV function was also severely reduced, with moderate-to-severe tricuspid regurgitation and mildly increased pulmonary artery pressure (40 mm Hg). There was no pericardial effusion. / Transthoracic echocardiogram showed a normal left ventricular systolic function with an LVEF of 53%. Apex was noted to be akinetic while other segments were moving normally. There was no pericardial effusion	NR
mall pericardial effusion and non-specific bronchocentric ground-glass opacities in the left lower zone which was reported as being suspicious for COVID-19	a trivial anterior pericardial effusion with good biventricular function	
no anomaly in the thoracic and abdominal areas	a non-dilated LV and severe and diffuse LV hypokinesia LVEF 30%	no significant lesions
compromise of the lung parenchyma, and mixed opacities, mainly alveolar and ground-glass of peripheral and basal predominance, a classic pattern of pulmonary involvement in COVID-19	left ventricle with global hypokinesia, with severely reduced systolic function, an ejection fraction of 30%, without valvular heart disease, and mild pericardial effusion (2 mm)	NR

NR	estimated left ventricular ejection fraction (LVEF) of 10–15%, reduced right ventricular systolic function, and global dilatation of all 4 chambers [Figure 3]. The right ventricle, right atrium and left atrium were moderately dilated, compared to mild left ventricular dilation. Specific measurements included: left ventricular internal diameter during end diastole (LVIDd) 5.8 cm, left ventricular internal diameter during end systole (LVIDs) of 5.1 cm, interventricular septum thickness (IVS) 1.3 cm and a Pulmonary Artery Systolic Pressure of 18 mm Hg. No significant valvular pathology was identified aside from mild regurgitation of the mitral and tricuspid valves. A pericardial effusion was not visualized.	NR
NR	extreme concentric left ventricular (LV) hypertrophy (LVH) with a wall thickness of 2.0 cm, reduced biventricular function with LV ejection fraction (EF) of 20%, and a small pericardial effusion	NR
discarded lung infiltrates but showed signs of ileocolitis	biventricular dysfunction, with left ventricular ejection fraction (LVEF) of 30% due to diffuse hypokinesis, diastolic dysfunction and slight posterior pericardial effusion without tamponade signs.	coronary angiography discarded coronary artery injury

NR	left ventricular ejection fraction (LVEF) of 26-30% and mild mitral regurgitation	no significant lesions
NR	echocardiographic signs of left ventricular (LV) dysfunction (decreased global and regional longitudinal strain or reduced LV ejection fraction and increased LV end-diastolic diameter)	no
enlarged abdominal lymph nodes, wall thickening of the colon and polyserositis with pericardial and pleural effusions and ascites	transthoracic echocardiography revealed a severely impaired left ventricular cardiac function (left ventricular ejection fraction, LVEF, 25%	no

subpleural consolidation containing air bronchogram with a halo of ground-glass opacification in the left upper lobe	NR	no
Normal	preserved left ventricular (LV) and right ventricular<br>(RV) contractility and no regional wall motion abnormality or<br>myocardial hypertrophy.	no
Normal	normal<br>systolic function with no peri-<br>cardial effusion	
Extensive diffuse bilateral airspace consolidations and ground-glass opacities most pronounced in the right upper lobe and bilateral lower lobes, with areas of subpleural sparing. Trace bilateral pleural effusions and minimal coronary artery calcification were noted.	moderate diffuse hypokinesis with relative apical sparing and a left ventricular ejection fraction of 35% to 40%	no
NR	disclosed moderate concentric hypertrophy, diminished intraventricular volumes with preserved left ventricular ejection fraction without segmental abnormalities, and moderate pericardial effusion with no clear signs of hemodynamic deterioration<br><br>2hours after admission - showed severe biventricular failure and diffuse myocardial edema	no

severe pneumonitis with multiple ground-glass opacities with a confluent appearance mainly at the level of the lower lobes, the lateral segment of the middle lobe and the lower and posterior lingular segments of the right upper lobe	normal size and function of the left ventricle (LV) with mild pericardial effusion	no
NR	mild left ventricular (LV) dysfunction	no
NR	NR	no
diffuse bilateral ground-glass opacities suggesting severe COVID-19 and viral pneumonia that scattered across two lungs with mild pleural effusion and cardiomegaly	reduced left ventricular ejection fraction (LVEF = 15%), high pulmonary arterial pressure (PAP = 50 mmHg), mildly enlarged LV and moderate to severe LV dysfunction, mild diastolic dysfunction (grade 1),mild mitral valve regurgitation, and normal septal thicknes	no
minor coronary artery calcification with no obstructive coronary artery disease	severely impaired left ventricular ejection fraction (LVEF) of 20% and mitral regurgitation	no

covid 19 pneumopathy with 50% of lung damage and bilateral pleural effusion	akinesia of the tip of the antero-septal and inferior wall of the left ventricular (LV) with severe hypokinesia of the inferior wall, a systolic dysfunction LV with an ejection fraction of 40%.	Performe d, without lesions.
A CT of the neck was performed to assess the neck swelling. This showed subcutaneous oedema on the left side with multiple enlarged lymph nodes up to 2.0×2.7 cm in diameter. The CT angiogram showed no coronary artery stenosis.	good ventricular function without hypokinesia and no significant valve pathology at admission. Echocardiography was repeated on day 2 revealing a deterioration of the left ventricular function with reduced systolic function of 40%	NR
NR	impaired left ventricular (LV) systolic function (ejection fraction [LVEF], 53%, end diastolic and systolic dimensions [LVDD and LVDS]: 42 and 31 mm, respectively) with thickened walls (interventricular septum [IVS], 12 mm; LV posterior wall [LVPW),], 12 mm), and mild pericardial effusion	normal coronary arteries
NR	severely depressed left ventricular function due to moderate to severe hypokinesia	

Pulmonary embolism was excluded based on the angio-CT. CT scan of the abdomen and pelvis with contrast revealed no focal changes of oncological concern or other enlarged pathological lymph nodes, but enlarged, homogenous liver without signs of cholestasis and an enlarged spleen.	globally reduced myocardial contractility and decreased ejection fraction up to 40%	NR
NR	left ventricular ejection fraction (LVEF) of 40–45%, mildly decreased globular left ventricular systolic function and moderate to severe hypokinesis involving inferior, inferoposterior wall without thinning of myocardium	no significant lesions
head CT without contrast was negative for ischemia or intracranial haemorrhage	new findings of severely reduced left ventricular (LV) systolic function, with an estimated ejection fraction (EF) of 20%. The patient had multiple LV wall abnormalities including akinetic inferolateral and apical anterior walls and hypokinetic basal and septal walls. A small pericardial effusion was also noted. Initial echocardiogram in (A) 4-chamber view shows multiple segmental abnormalities and an ejection fraction of 20%. (B) Parasternal long axis view shows multiple segmental abnormalities, trace effusion, andlow ejection fraction of 20%.	NR

pulmonary embolism	significant reduction in the left ventricu- lar ejection fraction to 25% with diffusely hypokinetic walls and a reduction in right ventricular function.	NR
NR	severe global left ventricular systolic dysfunction, RV enlargment, RV systolic dysfunction, modarate to large pericardial effusion, Intermittent RV impaired filling and collapse (s/o tamponade)	normal coronary arteries
moderate bilateral pleural effusion	decrease in left ventricular ejection fraction at 45%, low cardiac output (3 L/min), and both subtle hypertrophy and akinesia of posterolateral left ventricular wall with small pericardial effusion opposite.	normal coronary arteries
bilateral, patchy peripheral ground-glass opacities with a crazy-paving pattern, focal consolidations, and mild pleural effusion	highly reduced ventricular function [left ventricular ejection fraction (LVEF) 22%, right ventricular ejection fraction (RVEF) 28%	no
pneumonia	hyperdynamic ventricular function, although under massive support of inotropic agents and vasopressors.	no
NR	enlarged heart with a marked decrease in ventricular systolic function and an ejection fraction of 20%	no

negative for pulmonary embolism, clear lungs with any typical characteristics of COVID-19	LVEF 55-60%, no wall motion abnormalities, no evidence of pericarditis or pericardial effusion	normal coronary arteries
NR	NR	NR
Diffuse bilateral interstitial inflammation with sub-pleural consolidations.	LVEF 34%, Dilated LV (LVEDD 56mm), severe and diffuse LV hypokinesia	Unremarkable

MR findings
subepicardial late gadolinium enhancement of the apex and inferolateral wall
mild systolic dysfunction (LVEF = 49%) with discrete global hypokinesis, pericardial effusion localised mainly around the left ventricular latelar wall (~10mm), diffuse interstitial myocardial edema with an increased T2 signal intensity ratio, prolonged T1 relaxation times
diffuse high signal intensity in the LV myocardium, myocardial wall thickening (which suggested myocardial wall edema), extensive transmural late gadolinium enhancment
recovery of systolic function (day-7), with the persistence of mild hypokinesia at basal and mid left ventricular segments, diffuse myocardial edema, determining wall pseudo-hypertrophy,
NR

focal infero-basal LV wall edema, elevated T1 and T2 myocardial relaxation times, the post-contrast sequences showed sub-epicardial and mid-wall late gadolinium enhancement in the basal inferior, basal infero-lateral, and anterior LV walls

LVEF 22%, late gadolinium enhancement images showed a pattern of extensive subepicardial and several midwall subepicardial spots of enhancement of the left ventricle, myocardial edema was absent (post-myocarditis state without active myocarditis)

increased wall thickness with diffuse biventricular hypokinesis, especially in the apical segments, severe LV dysfunction (LVEF 35%), biventricular myocardial interstitial oedema, Phase-sensitive inversion recovery sequences showed diffuse late gadolinium enhancement extended to the entire biventricular wall, pericardial effusion was confirmed,

NR

NR

NR

NR

NR

NR

NR

NR

NR

NR

NR

NR

Cardiac magnetic resonance (CMR or cardiac MRI) imaging showed myocardial edema and hyperemia suggestive of active myocarditis

NR

NR

NR

NR

NR

-

extensive subepicardial
fibrosis with diffuse contrast uptake at the level of the pericardium

Cardiac magnetic resonance imaging showed an area of myocardial late enhancement compatible with the diagnosis of myocarditis

NR

T2 sequences showed diffuse hyperintense myocardium but suffered from too many artefacts to be considered diagnostic quality. Steady-state free precession cine images demonstrated a biventricular cardiomyopathy (left ventricular ejection fraction: 25%) with myocardial wall thickening. First-pass contrast-enhanced CMR did not reveal subendocardial perfusion defect. Late gadolinium enhancement images demonstrated massive, heterogeneous, and predominantly subepicardial enhancement of the left ventricular myocardium indicating a severe inflammation, without evidence for ischaemic heart disease

NR/ normal biventricular volumes and function. There was akinesia of the apico-lateral, apico-inferior walls and apical cap, myocardial hyperemia, and myocardial scar/necrosis consistent with a diagnosis of myocarditis

n MRI of both lower limbs confirmed features of diffuse myositis with symmetrical appearances involving the anterior, medial and posterior muscle compartments of the thighs with subcutaneous oedema. . A cardiac MRI showed normal left and right ventricular function, with possible evidence of myocardial oedema in the basal inferoseptum, in keeping with myocarditis, as well as a 16 mm circumferential pericardial effusion

NR

cardiac magnetic resonance imaging (MRI) was performed , which showed inflammatory manifestations, with the recovery of the ejection fraction

NR

NR

NR

myocardial necrosis, fibrosis, and hyperemia, indicating myocarditis according to the Lake Louise criteria

patient A—native T1 map showing prolonged T1 relaxation times in the posterior interventricular septum and corresponding late gadolinium enhancement image (LGE) with enhancement in the posterior septum (arrowhead), consistent with acute myocarditis. Transthoracic echocardiography showed a preserved left ventricular (LV) systolic function (EF 60%) without any wall motion abnormalities, but focal echo-bright appearance of the interventricular septum (not shown) and slightly impaired global longitudinal strain. The two right panels: patient B—representative cMRI scans of the patient who had a history of coronary artery disease treated by percutaneous coronary intervention (everolimus-eluting stent in the right coronary artery). T2-short TI inversion recovery image, showing diffuse myocardial oedema and LGE image with subtle subepicardial enhancement of the lateral wall (arrowheads).

NR

Left ventricular function, volumes, and mass were in normal
range (ejection fraction 64%, stroke volume 62.2 mL, end-diastolic vol-
ume 97 mL, end-systolic volume 34.8 mL, mass 128 g) (Video 1). T2
short tau inversion recovery (STIR) sequence revealed a subepicardial
high signal intensity in the mid posterolateral wall of the left ventricle
which suggests myocardial wall edema (Fig. 2A). Cardiac MRI showed
subepicardial late gadolinium enhancement of the posterolateral wall
in the mid ventricle-suggestive of myocarditis at 5 and 10 min after con-
trast administration, respectively

The CMR scan confirmed normal LV and RV function and wall thickness with an LV ejection fraction of 65%, RV ejection fraction of 56% and wall thickness at end-diastole of 7 mm. There was delayed late gadolinium enhancement (LGE) within the mid-wall as well as the epicardial regions involving the LV basal inferolateral wall, mid-anterolateral and mid- inferolateral wall. T2 short tau inversion recovery (STIR) black blood imaging showed high signal intensity within the LV anterolateral, inferolateral wall at the base and mid-ventricle level. On non-contrast T1 mapping, there was a prolongation of T1 time at the mid-ventricle segment 5 (1212 ± 141 ms) and segment 6 (1113 ± 93.8 milliseconds).

late subepicardial enhancement predominating in the inferior and lateral wall

diffuse biventricular and
biatrial edema with a small area of late gadolinium
enhancement (LGE)

NR

confirmed normal LV dimensions and function (LV
ejection fraction of 59%) and revealed the presence of right and left apical
microaneurysms. Late gadolinium enhancement
(LGE) imaging showed a subepicardial area in the infero-lateral wall on basal and mid-
ventricular planes; corresponding mapping sequences documented a focal
increase in native T1 values, extracellular volume fraction and T2
mapping values consistent with diffuse edema and combined extracellular
matrix expansion

normal LV size with mildly reduced ejection fraction
(LVEF) of 50%; T2-weighted oedema sequence with its post-analysis T2 ratio showed
oedema/inflammation in the mid inferoseptal and inferior wall. Late gadolinium
enhancement showed subepicardial
fibrosis in the mid inferior wall

left atrial enlargement and global left ventricular hypokinesia with ejection fraction of
20%. The T2-weighted sequence did not show myocardial edema. In contrast, imaging
with the use of late gadolinium enhancement demonstrated a large, patchy, and linear
nonischemic pattern of fibrosis localized subepicardially and intramurally in the basal
and midcavity segments of the inferior and inferolateral wall and in the apical segments
of the inferior wall

no

biventricular dysfunction with an LVEF
of 30%. STIR sequences showed biventricular oedema and reduced myocardial T1

focal hypertrophy in the antero-septal and inferior segment of the LV extended over approximately 62mm, and in antero-inferior of the RV extended over 27mm, in hyper signal T2, with an intense enhancement on the tardive injected sequences compared to the rest of the myocardium in favor of a myocarditis

diffuse myocardial oedema

high signal of T2-weighted black blood in
the anterior, interventricular septal, and posterolateral walls of the LV (A, short-axis)
and late gadolinium enhancement in the posterolateral wall of the LV

Minimal pericardial effusion and contrast enhancement of the pericardium but
not the myocardium were seen on magnetic resonance imaging
scan

NR

mild to moderately reduced LVEF (38%); moderate hypokinesis of midventricular inferolateral wall; edema/inflammation of epicardium and epicardial scar of basal anteroseptal and anterior wall and midventricular anterolateral, inferolateral and inferior wall

NR

NR

NR

normal left ventricular size (left ventricular end-diastolic volume index: 75 mL/m2) and mild systolic dys- function (left ventricular ejection fraction: 45%) with global hypokinesia. The presence of myocardial inflammation was confirmed by T2 mapping (global T2 relaxation times: 62 ms; centre-specific cut-off value for acute myocarditis: ≥55 ms). Late gadolinium enhancement imaging (inversion time by using the Look-Locker technique: 280 ms) showed focal lateral subepicardial enhancement with prolonged T1 relaxation times (global T1 relaxation times: 1160 ms; centre-specific cut-off value for acute myocarditis: ≥1000 ms). Cardiac magnetic resonance imaging also showed small pericardial effusion.

late gadolinium enhancement (LGE) of the entire left ventricular myocardium with intracardial thrombi was detected on MRI (Figure 1B–K). T1 and T2 times were markedly prolonged, reflecting acute oedema following myocardial inflammatio

no

no

not performed

right pulmonary condensation along with associated pleural effusion, LV of normal volume with concentric hypertrophy and mild depressed systolic function (LVEF 53%) secondary to diffuse hypokinesia was observed. Global longitudinal strain was decreased. Myocardial oedema with a predominant subepicardial pattern was seen in the lateral, anterior, inferior, and apical segments on T2 short tau inversion recovery and T2 mapping sequences, determining wall pseudo-hypertrophy. Pericardial oedema without associated effusion was also observed. Native T1 and extracellular volume both were increased in relation to the acute inflammatory process. Late gadolinium sequences showed extensive, patchy intramyocardial/subepicardial enhancement affecting the entire lateral, anterior, inferior, and apical septal walls and the pericardium.

NR

Lake-Louis Criteria (yes/no)	Biopsy findings
yes	
yes	
yes	
yes	diffuse T-lymphocytic inflammatory infiltrates (CD3+ >7/mm^2) with huge interstitial edema and limited foci of necrosis
	myocarditis with inflammatory infiltrates consisting of a majority of lymphocytes and neutrophils, oedema but not typical myocyte necrosis, CD-4-positive T lymphocytes, RT-PCR for SARS-CoV-2 and PCR for other cardiotropic viruses was negative

	diffuse lymphocyte infiltration in the interstitium of the myocardial tissue, atrophy and shedding degeneration myofibrils, no myocardial necrosis
yes	NR
no	focal subendocardial fibrosis and no signs of clinical relevant rejection
yes	NR
no	NR
	NR

NR	eosinophilic inflitrate of the myocardium (infiltrate consisting mainly of mononuclear cells - few lymphocytes and histiocytes with many eosynophiles)
NR	mixed inflammatory infiltrate with lymphocytes, histiocytes, neutrophils, eosynophiles
NR	mild lymph-histiocytic interstitial myocardial infiltrate with occasional neutrophil
NR/ no	NR
NR	NR

NR	NR
NR	NR
NR	NR
NR	NR
NR	NR

yes	NR
NR	multifocal inflammatory infiltrates consisting of neutrophilic granulocytes, lymphocytes and histiocytes, capillarostasis, and perifocal single-cell necroses of cardiomyocytes
no	no
no	NR
no	Mild lymphocytic infiltration and moderate to severe perivascular fibrosis with wall thickening of intramural arterioles. There was no sign of severe myocardial injury compatible with typical active myocarditis. However, ischemic changes were found with a focal coagulative necrotic area at microvascular level (approximately 0.08 mm2 in area) with losing nuclei, accompanied by microthrombi with fibrin and platelets in small vessels. Scattered megakaryocytes were also seen in the capillaries. Microthrombi was seen throughout the specimens and myocytes in non-necrotic areas often showed diffuse cytoplasmic vacuolization. The presence of platelets in obstructive and non-obstructive microthrombi within the lumens of microvessels was confirmed by immunohistochemical expression of CD61. Fibrin-rich microthrombi were also present as confirmed by phosphotungstic acid-hematoxylin (PTAH) stain. Microvessels including intramural arterioles often showed swollen endothelial cells.

no	diffuse interstitial and perivascular neutrophilic and lymphocytic infiltration with rare eosinophils and rare myocyte necrosis, suggesting fulminant myocarditis
no	A subtle mild interstitial infiltrate consisting mostly of CD68+ macrophages along with a lesser number of CD3+ T cells. Microthrombi were identified, with the degradation products present suggestive of a sub-acute microangiopathic process. No fibrosis nor myocardial necrosis was present. No viral particles were seen on electron microscopy. With regard to other causes of myocarditis, the EMB did not show interstitial fibrosis, iron accumulation, evidence of sarcoid or amyloid, or changes consistent with hypertrophic obstructive cardiomyopathy.
no	NR
no	slight interstitial fibrosis without inflammation. Immunohistochemical staining (CD3, CD20, and CD68) confirmed the absence of interstitial inflammation

no	no
yes	explantated heart, with large areas of myocardial necrosis, suppurated lesions, and lymphocytic infiltration, polymorphic inflammatory infiltrate, mostly in myocardium areas but focally extended to endocardium and pericardium. Lymphocytes were predominant. Infiltrate also contained plasmocytes, neutrophils, eosinophils, and histiocytic and giant cells. Clusters of neutrophils with leucocytoclasia suggested suppurated lesions. Myocardial cells were suffering with clarified cytoplasm, sometimes fibrillary or eosinophil, and with enlarged dystrophic nucleus. Large areas of necrosis and haemorrhage were seen among these inflammatory areas. Adjacent myocardium showed interstitial oedema and focally recent interstitial fibrosis. Ziehl, Grocott, Gram, and EBER ISH stains were negative. Most of lymphocytes were T phenotype CD5+, with only a few associated B lymphocytes CD20+. Numerous histiocytic cells were revealed by CD163 immunostaining. Cytomegalovirus immunostaining was negative as well as Epstein–Barr virus in situ hybridization. A (×2) and B (×20): necrosis and polymorph inflammatory infiltrate with haematic suffusion in endocardium and myocardium. C (×10) and D (×20): pericardial inflammatory reaction.

NR/ no	NR
no	NR
NR	Biopsy after death - Microscopic examination of the heart revealed the existence of a multifocal inflammatory infiltration, in both ventricles and septum, composed in its majority of macrophages and lymphocytes (Fig. 1), associated with a mild polymorphonuclear neutrophils infiltrate. The myocardium was edematous, containing dystrophic cardiomyocytes, without necrosis. An hypocellular confluent area of fibrosis was found in posterior wall of left ventricle. There was no significant inflammatory infiltrate in the wall of capillaries and vessels.
no	patient did not consent

NR

NR

NR

yes	NR
yes	Immunohistochemistry and histology revealed myocardial inflammation in the absence of cardiomyocyte necrosis (Dallas criteria of ‘borderline myocarditis’6), with increased lymphocytes (CD3, LFA-1, and CD45R0) and macrophages (Mac-1), in part with highly abundant perforin-positive cytotoxic T cells. The inflammatory process seemed to be paralleled by increased thickness of small arteries
no	EMB showed a significant infiltration of immune cells into the heart. Especially CD68+ macrophages but also CD3+ T cells were found to be located primarily around small vessels within the myocardium, as shown by immunohistochemical stainings. Masson Trichrome and HE stainings further demonstrated the presence of perivascular fibrosis in serial tissue sections, but no myocyte necrosis

yes	no
yes	no
yes?	no
yes	no
no	no

yes	diffusely mononuclear infiltrates associated with necrosis of adjacent myocytes<br>and necrotizing vasculitis of intramural coronary arteries associated to positivity of CD45RO+ T-lymphocytes of the affected vessel wall. Arterioles<br>not infiltrated by inflammatory cells presented at immunohistochemistry complement frac-<br>tions (C3d) deposition suggesting coexisting endothelitis. Moreover, real time<br>PCR on two frozen endomyocardial samples for SARS-COV-2, and for the most common<br>cardiotropic viruses including adenovirus, cytomegalovirus, parvovirus B19, Epstein–Barr<br>virus, human herpes virus 6, and herpes simplex virus 1 and 2, enterovirus, influenza virus<br>A H1N1 and B and hepatitis C virus was negative
yes	not performed
no	not performed
no	not performed
no	not performed

yes	not performed
no	not performed
yes	infiltration of interstitial mononuclear cells (Fig. 3). Infiltrating cells were<br>mainly T-cells and macrophages, with more CD4-positive<br>cells than CD8-positive cells, and few B-cells (Fig. 3). RTPCR SARS-Cov-2<br>was negative in the myocardium.<br>Electron microscopy revealed inflammatory cell infiltration and<br>myocyte damage compatible with myocarditis<br>(Fig. 4). However, viral particles were not detected in the<br>high-power fields.<br>Tests for viral antibodies (using paired serum samples)<br>against adenovirus, coxsackie virus (A16, A7, B1, B2, B3,<br>B4, B5, and B6), echovirus (3, 6, 7, 11, and 12), and<br>parainfluenza virus (1, 2, and 3) were all negative
no	mononuclear<br>infiltration of the myocardium, predominantly with T lymphocytes and<br>macrophages, Myocardial biopsy shows mononuclear infiltration of<br>the myocardium, with<br>CD3-positive lymphocytes (arrow) and CD68-positive macrophages<br>(arrow), without formation of granulomas

NR	NR
NR	not performed
NR	NR

NR	NR
NR	NR
yes	multiple foci of lymphocytes in a diffuse inflammatory and oedematous back- ground.
yes	active lymphocytic myocarditis
no	Slightly diffuse interstitial mononuclear inflammatory in- filtrates, dominated by lymphocytes. No thrombotic events were observed in the microcirculation of the heart. In the patchy areas, the lymphocytes ‘interlocked’ the myocytes, resulting in myocyte degeneration and necrosis (piecemeal necrosis). Intermingled with lymphocytes, only a very few individual polymorphous neutrophils were found in the affected areas. Signs of inflammation were present in both the epicardium and the myocardium.
no	not performed

no	not performed
yes	NR
no	Low-grade interstitial and endocardial inflammation. Large, vacuolated, CD-68-positive macrophages were seen with immunelight microscopy. The ultrastructural study demonstrated single or small groups of viral particles with the morphology and size of coronaviruses. The viral particles were observed in cytopathic, structurally damaged interstitial cells that demonstrated loss of cytoplasmic membrane integrity. Cardiac myocytes showed non-specific features of local myofibrillar lysis and lipid droplets. Interstitial fibrosis was minimal, focal, and mainly perivascular.

Dallas criteria (yes/no)
yes
yes

yes

no

no

NR

no

yes

yes

yes

no

NR

NR

NR

NR

NR

NR

NR

yes

no

no

yes

yes

yes

no

no

no

yes

yes

yes

yes

no

no

no

no

no

yes

No

no

no

no

NR

NR

NR

NR

NR

NR

yes

yes

yes

no

no

no

yes

ESC Criteria (I) ECG/Holter/stress test (II)cardiac injury markers (TnT/TnI) (III) abnormalities on cardiac imaging (ECHO/angio/CMR) (IV) tissue charakterization by CMR
I, II, III
II, III, IV
II, III, IV
I, II, III, IV,

I, II,

III, IV

II, III, IV

I, II, III, IV,

I, II,

II, III

I, II, III

II, III

II, III

II, III

II, III

I, II,III

II, III

II,III

I, II, III

I, II, III

II, III, IV

I, II

I, II, III

I, II, III

II

I, II, III

II, III

III, IV

I, II, III, IV

II, III

I, II,III, IV

II, III/ I, II, III, IV

I, II, III

II,

I, II, III

II, III

II, III

II,III

I, II, III. IV

I,II,III, IV

II, III

II, III, IV

I, II, IV

IV

II, III, IV

I, II, III

I, II, IV

III, IV

II,III

II, III

II, III

I,II, III, IV

I,II,III,IV

II,III

II,III

I, II, III

I, II, III

I, II, III

I, II, III

II, III

II, III, IV

I, II,

I, II, III

I, II

I, II, III, IV

II, III

ESC criteria summary ex. I, II, III = 3 (number)	Managment and outcome
3	
3	
3	
4	

2

2

3

4

2

2

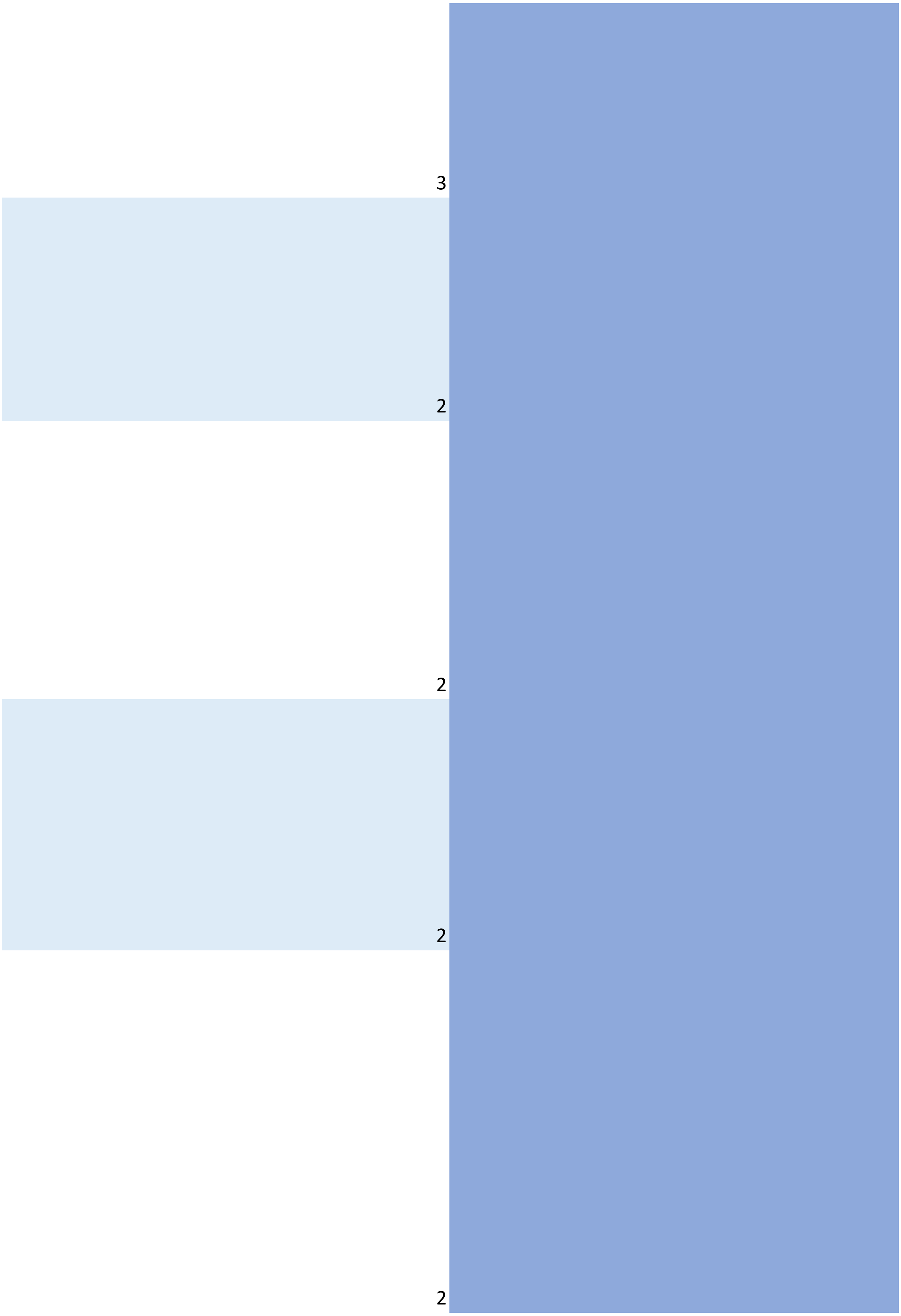
3
2
2
2
2

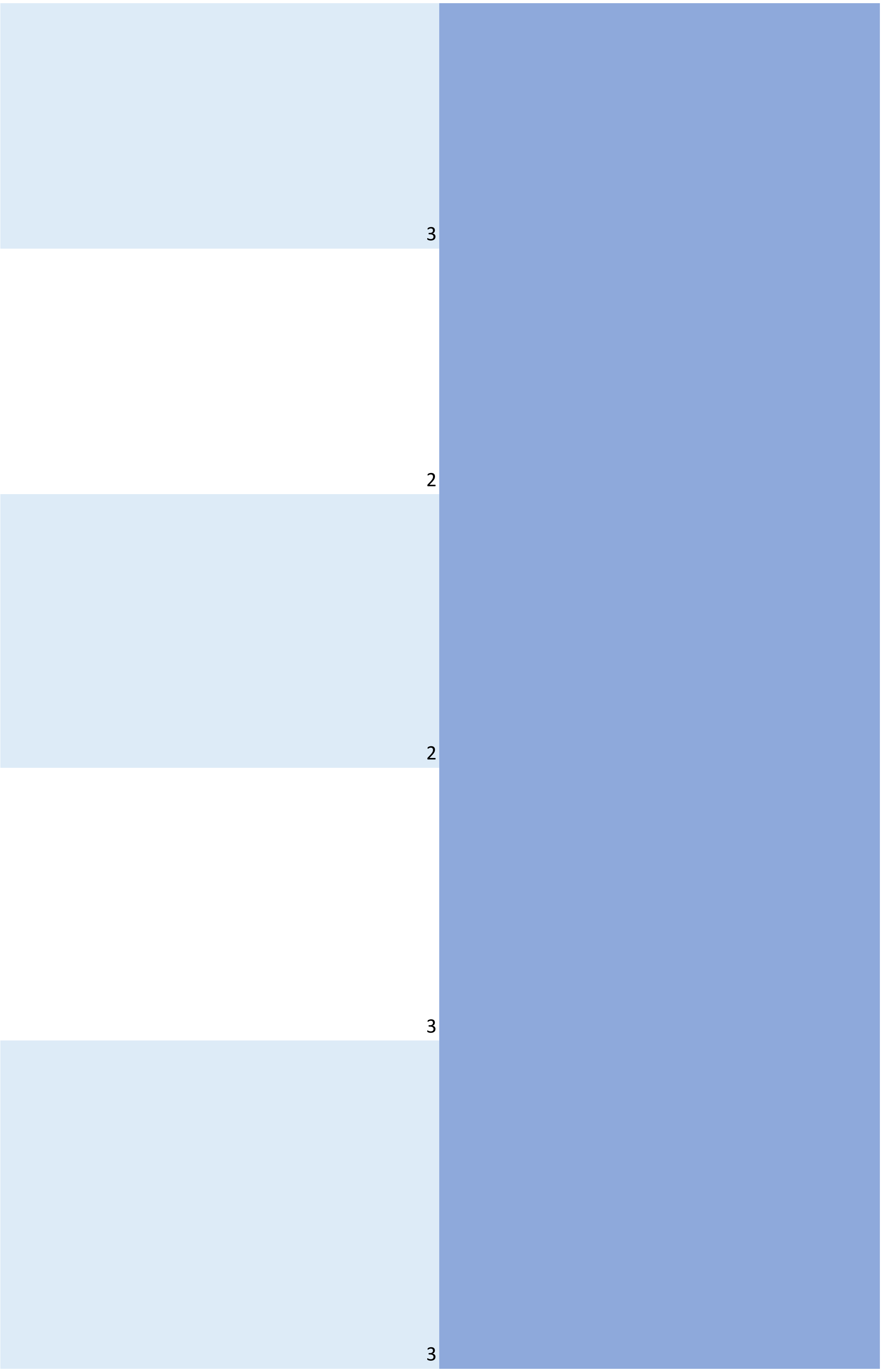
3
2
2
3
3

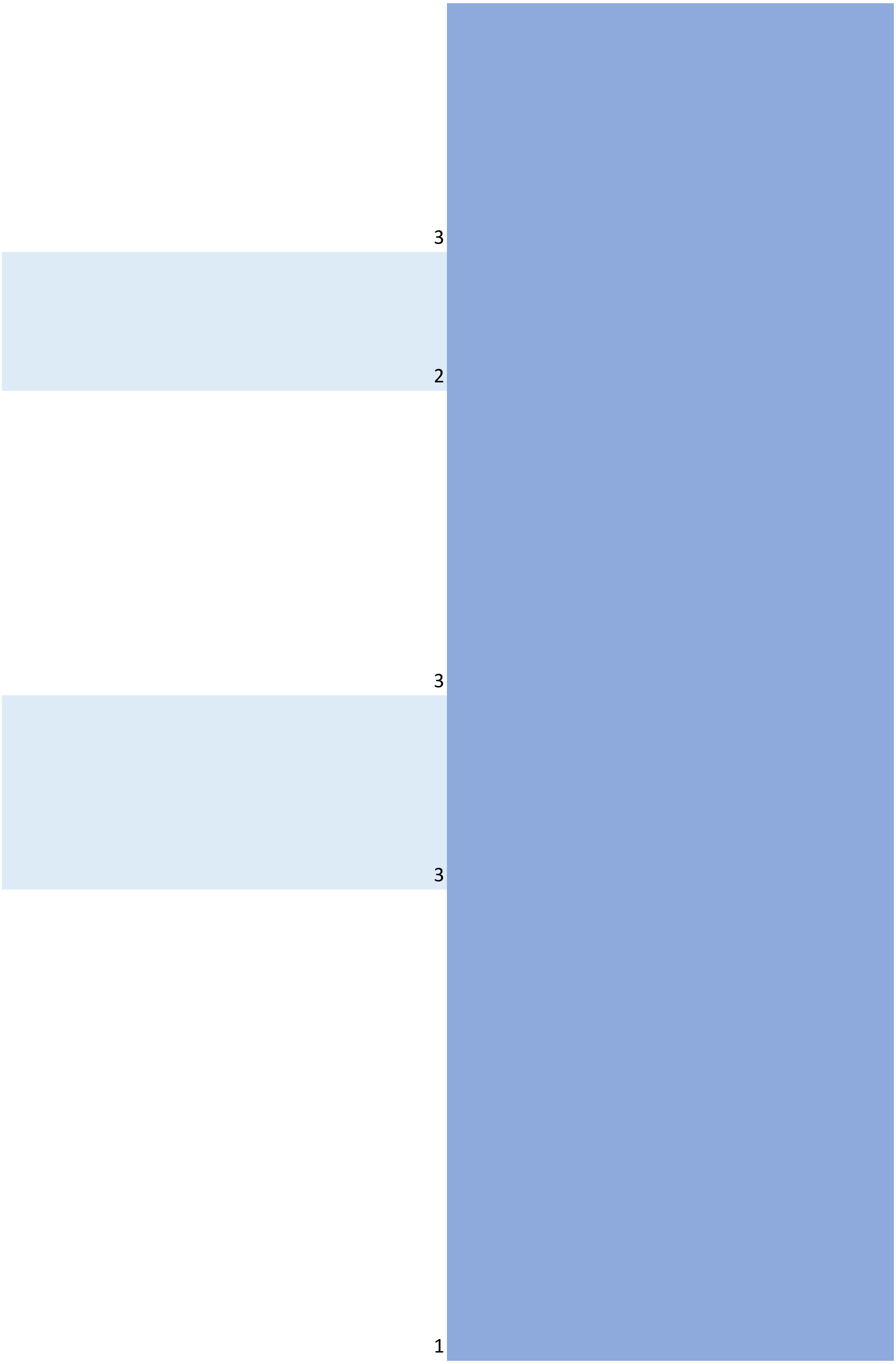
3
2
3
3
1

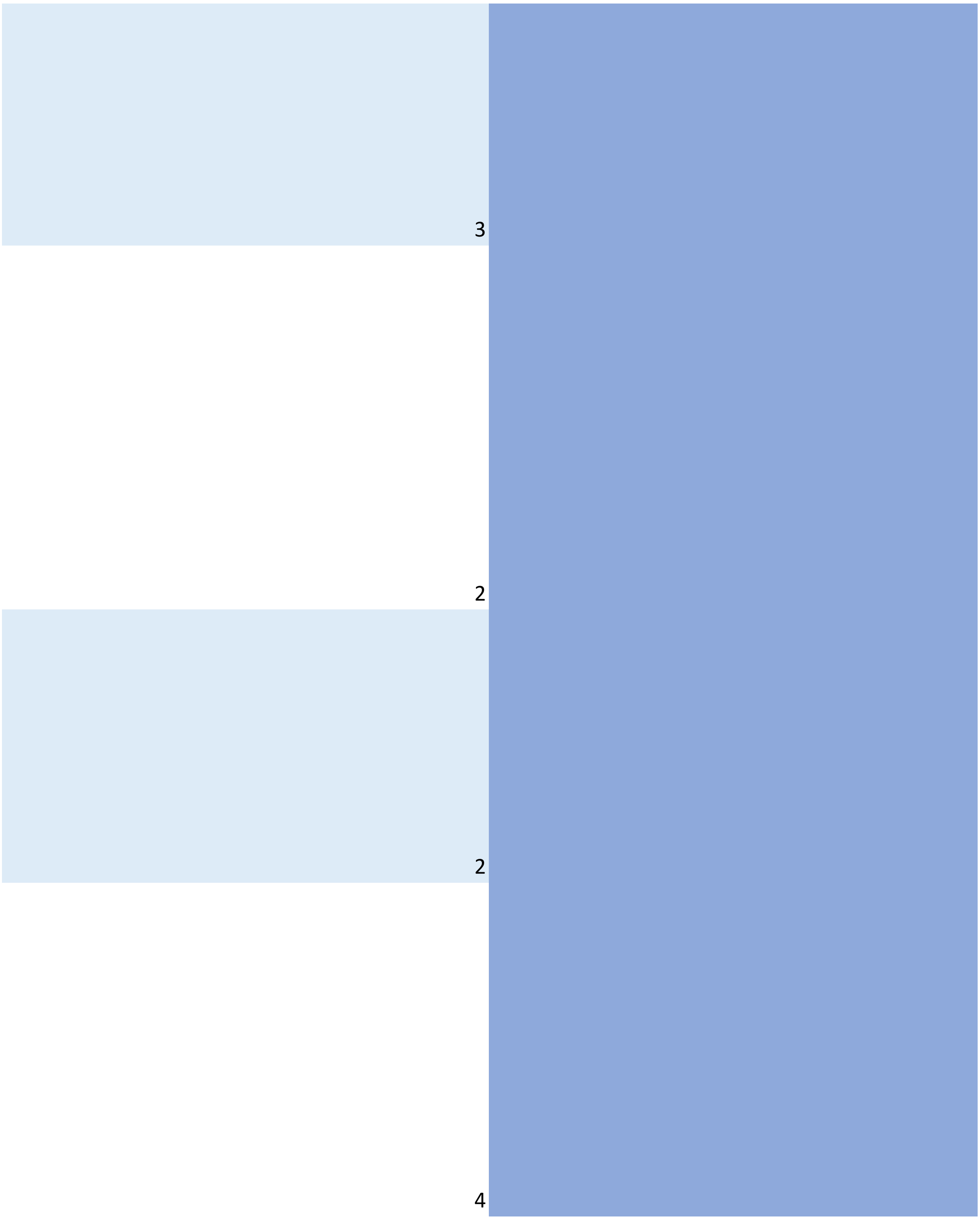
3
2
2
4

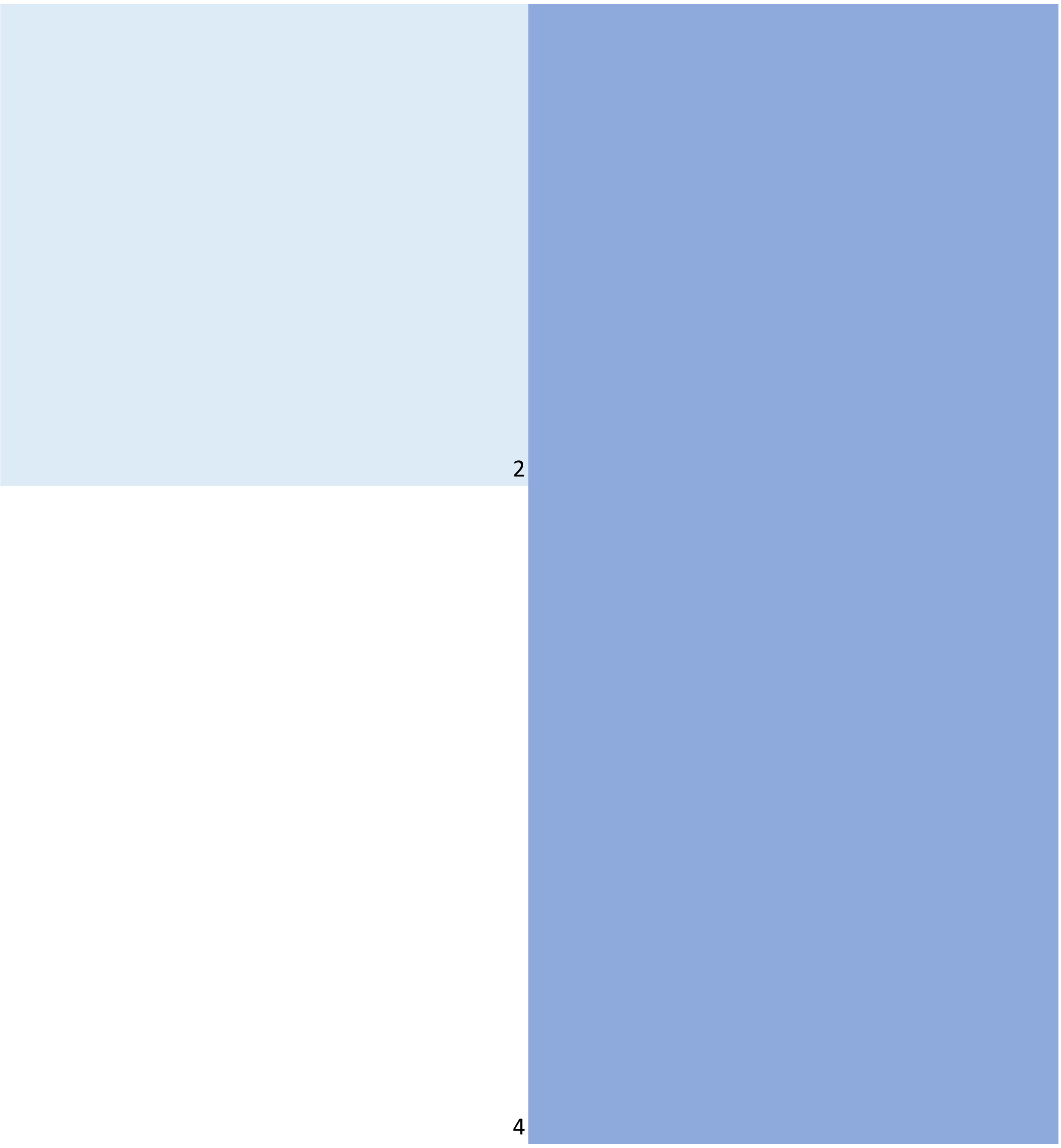
2
4

2 and 4

3

1

3

2

2

2

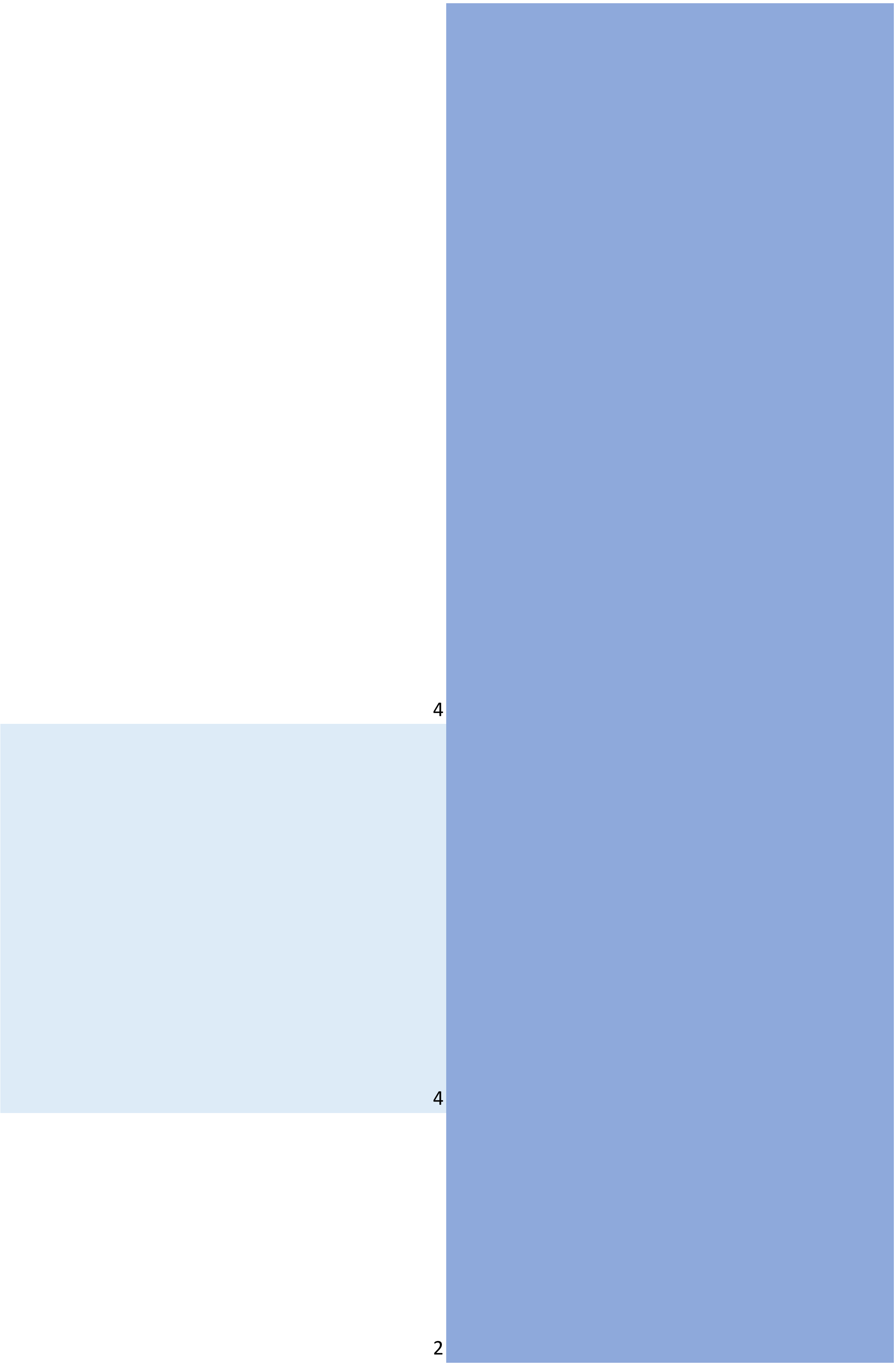
4
4
2

3

3

1

3

3

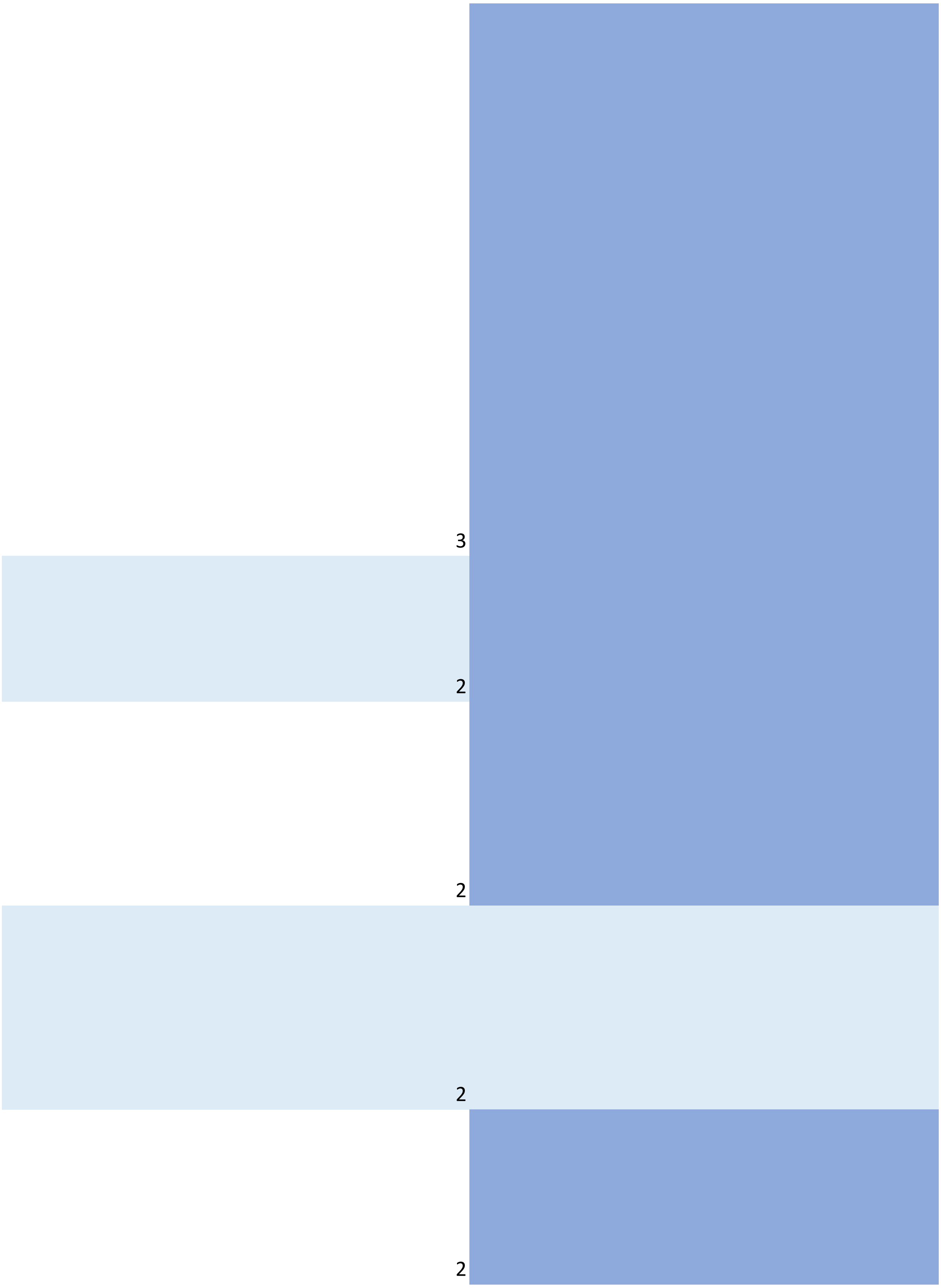
3
2
2
2
2

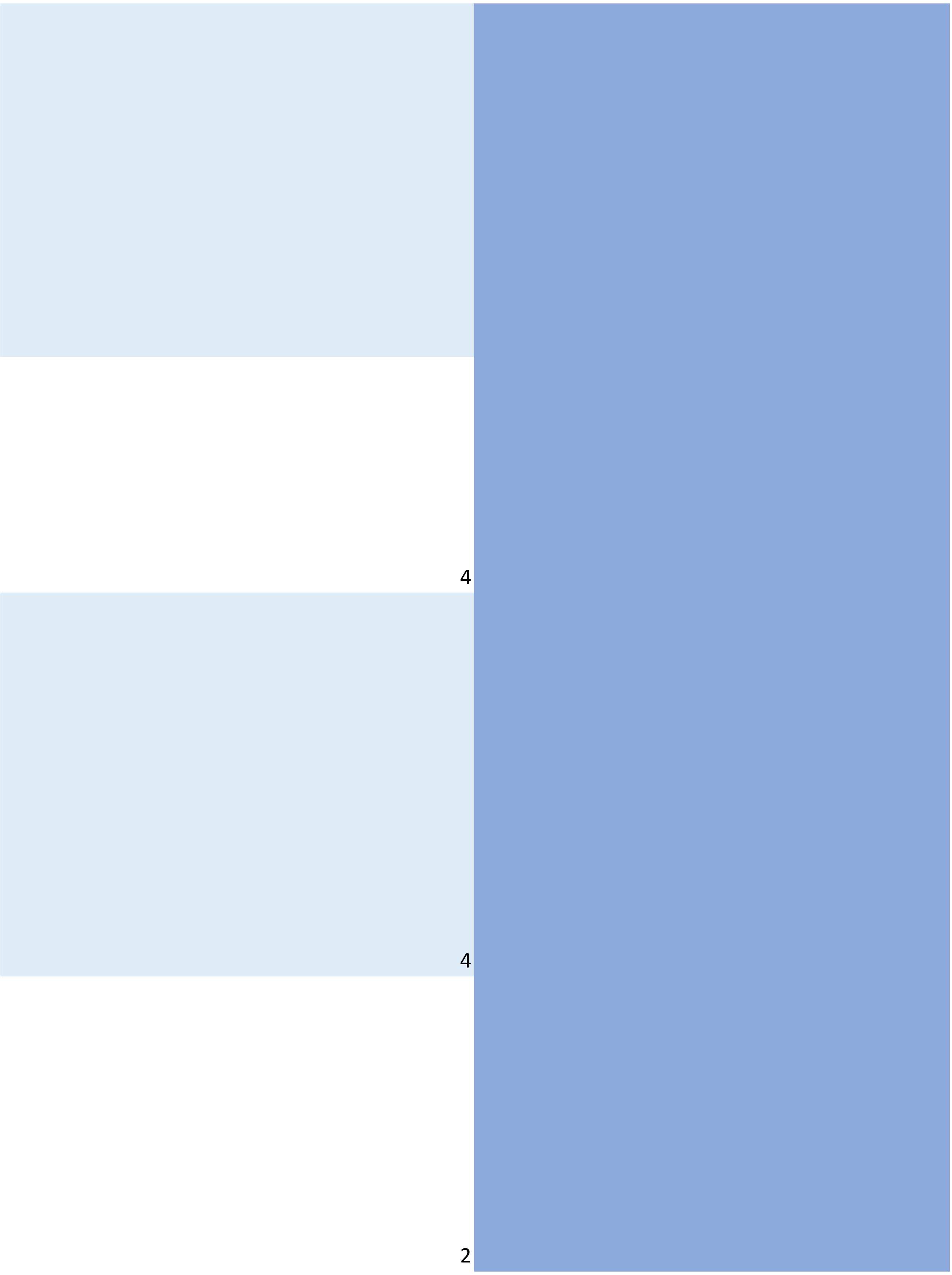
4
4
2

2

3

3

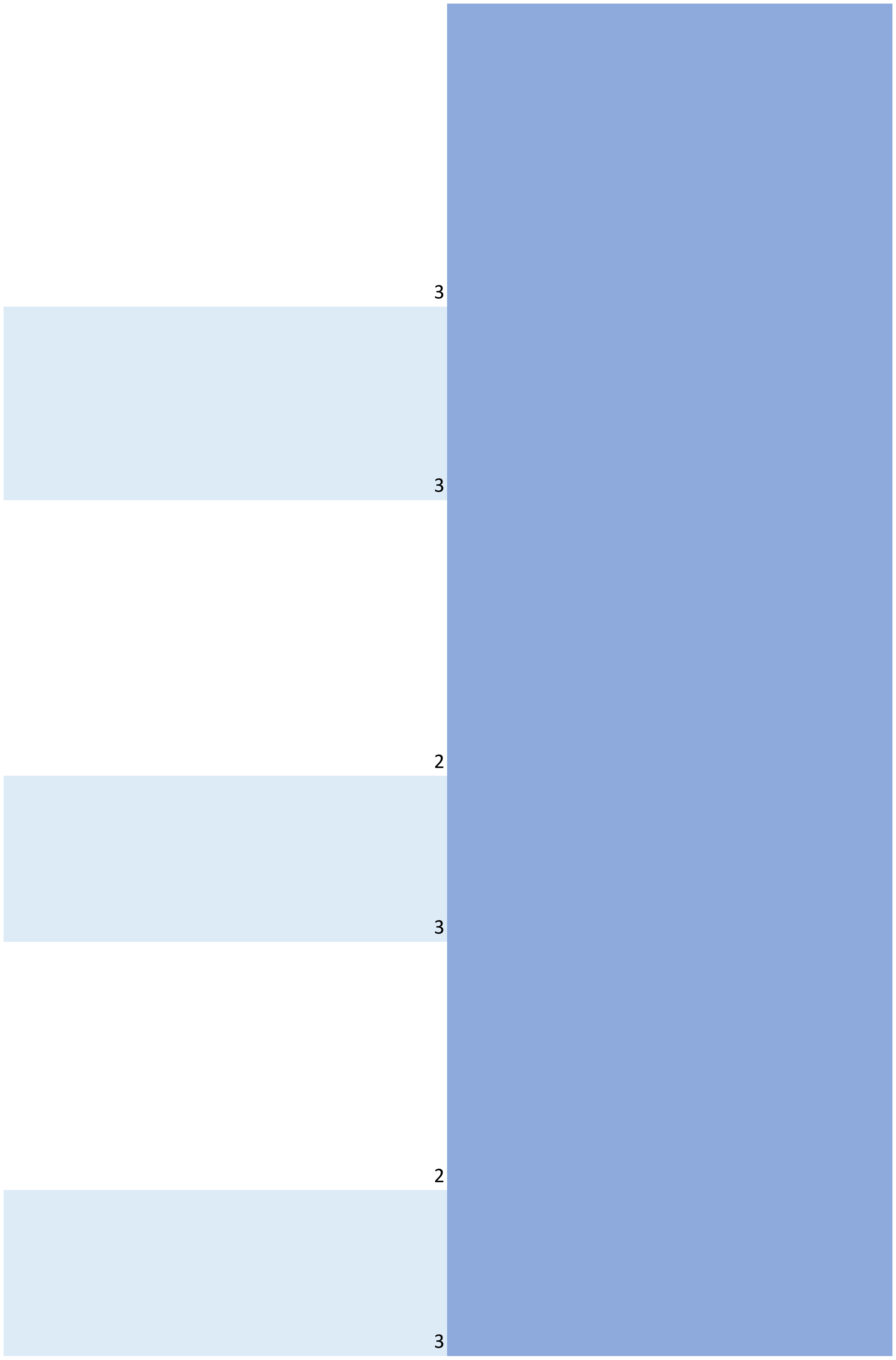
3
3
2
3
2
3

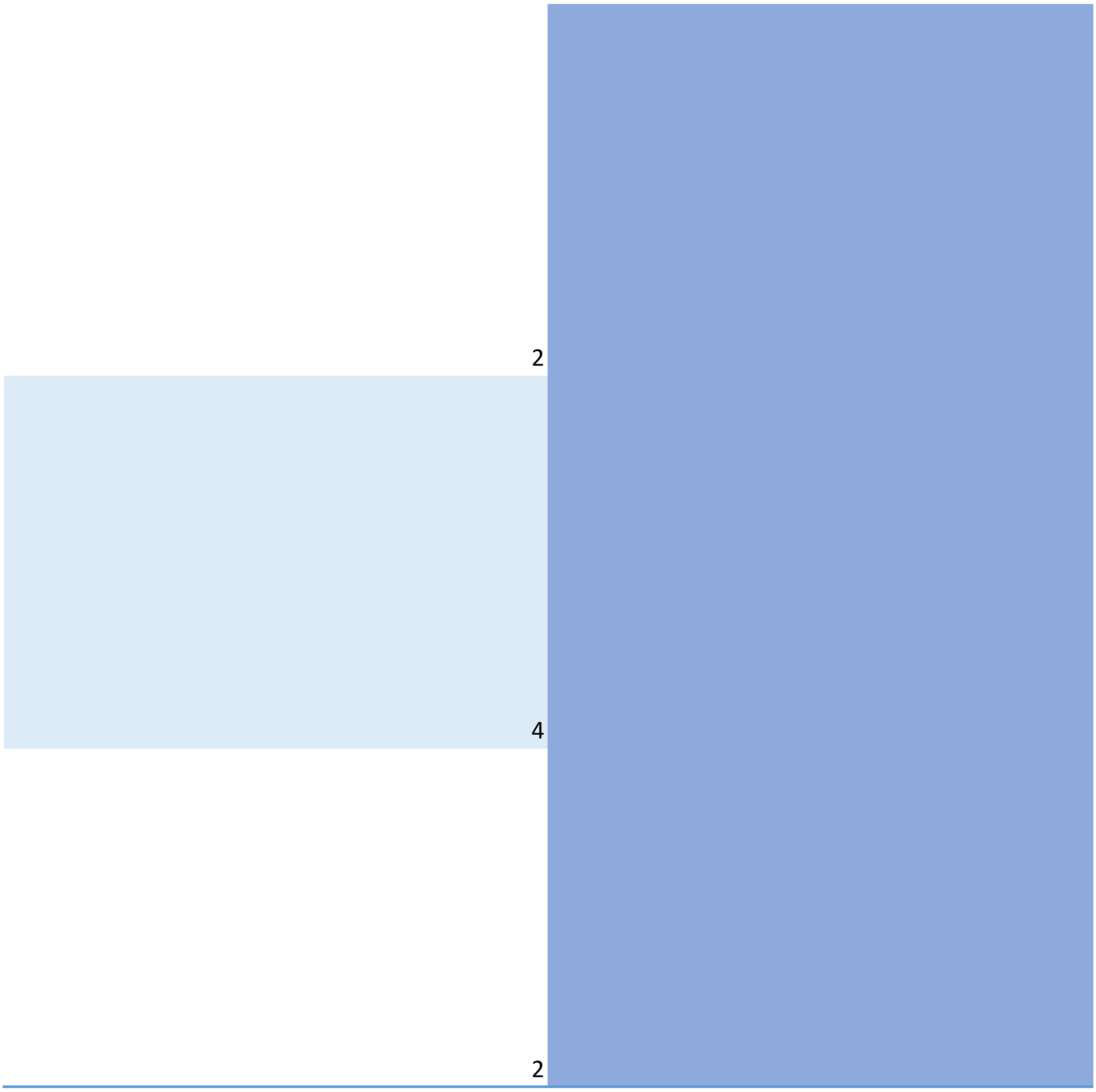
2
4
2

In-hospital mangment (pharmacotherapy)	In-hospital managment (procedures - ECMO, intubation, tranplant etc.)
aspirin, fondaparinux, hydrocortisone	mechanical ventilation
NR	NR
NR	NR
lopinavir/ritonavir, hydroxychloroquine,	CPAP
high-dose diuretics, broad-spectrum antibiotics, inotropic and vasopressive support	mechanical ventilation,

immunoglobulins, steroids, anticoagulant (nafamostat metylate)	IABP, V-A ECMO, CRRT
bisoprolol, ramipril	NR
methylprednisolone 1g daily, intravenous diuretics	NR
dobutamine, kanrenone 50mg, furosemide 20-40mg, bisoprolol 2,5mg, aspirin 500mg twice a day, hydroxychloroquine 200mg twice a daily, lopinavir/ritonavir 2 tablets of 200/50mg twice a day, methylprednisolone 1mg/kg for 3 days,	NR
methylprednisolone 200mg/day, immunoglobulin 20g/day, norepinephrine, diuretics (furosemide and torasemide), milrinone, piperacillin sulbactam, pantoprazol	NR
lopinavir/ritonavir, interferon alfa-1b, methylprednisolone, immunoglobulin, piperacillin-tazobactam,	ventilatory support - high-flow oxygen, CRRT, ECMO,

IV methylprednisolone at 1.2mg/kg/d (40-mg Q8h), changed to high-dose IV methylprednisolone “pulse” therapy of 10mg/kg/d (1 g/d) for 3 days followed by tapering dose over 8 weeks. vasopresors: norepinephrine 20 mcg/min, dobutamine 10 µg/kg/min, and milrinone 0.5 mcg/kg/min	femoral-femoral VA-ECMO, CRRT, biventricular assist devices (BiVAD) with right ventricular assist device (RVAD) and Impella cardiac power (CP) microaxial left ventricular assist device (LVAD), pericardiostomy
vasopressors, inotropes, piperacillin/tazobactam, and vancomycin, norepinephrine 32 µg/min and epinephrin 5 µg/min, high-dose IV methylprednisolone “pulse” therapy of 15mg/kg/d (1g/d) for 3 days followed by tapering dose over 8 weeks	Impella CP microaxial LVAD, CRRT
high-dose corticosteroid pulse therapy, anakinra (interleukin-1 receptor antagonist) IV 100mg tid for 7 days and IV immunoglobulin (IVIG) 2-g/kg single dose, empirically on piperacillin/tazobactam and vancomycin, inotropic agents (milrinone 0.375 mcg/kg/ min and dobutamine 2.5 µg/kg/min), vasopressors (norepinephrine 20 mcg/min)	femoral-femoral VA-ECMO, Impella CP
intravenous immunoglobulin (IVIG; 1 g/kg), broad-spectrum antibiotics (meropenem [500 mg] and doxycycline [200 mg/day]), high-dose corticosteroid (methylprednisolone 1 g/day), and remdesivir for COVID-19	NR
corticosteroids (methylprednisolone 1 mg/kg/day), broad-spectrum antibiotics (meropenem [500 mg q8 h for 5 days] and doxycycline [200 mg/ day]), and remdesivir for COVID-19	NR

meropenem [500 mg q8 h for 5 days] and doxycycline [200 mg/day]), methylprednisolone 1 g/day, and remdesivir	intubation, DC shock
meropenem [500 mg q8 h for 5 days] and doxycycline<br>[200 mg/day]), methylprednisolone 1 g/day, and remdesivir	NR
meropenem [500 mg q8 h for 5 days] and doxycycline [200 mg/day]), corticosteroid (methylprednisolone 1 mg/kg/day), and remdesivir.	NR
broad-spectrum antibiotics (meropenem [500 mg q8 h for 5 days] and doxycycline [200 mg/day]), corticosteroid (methylprednisolone<br>1 mg/kg/day), and remdesivir	NR
IVIG (1 g/kg),broad-spectrum antibiotics (meropenem [500 mg q8 h for 5 days] and doxycycline [200 mg/day]), high-dose corticosteroid (methylprednisolone 1 g/day), and remdesivir	cardio-version and lidocaine administration, resuscitation

heparin, dexamethasone	NR
continuous adrenalin/noradrenaline infusion, prophylactic antibiotic therapy (piperacillin/tazobactam)	2xresuscitation
amoxiclav, then i.v. piperacillin–tazobactam.	oxygen requirement via nasal cannula.
a 4-day regimen of intravenous immunoglobulin (IVIG) 20g daily and methylprednisolone (50 mg every 6 h)	initially placed on a non-rebreather mask with supplemental oxygen via nasal cannula and placed in the prone position, then quickly intubated and started on hydroxychloroquine therapy
methylprednisolone (1000 mg daily for 3 days) and gamma globulin (80 g daily for 2 days) in addition to catecholamine infusion and ECMO support	intubated and cannulated for extracorporeal membrane oxygenation (ECMO)

intravenous fluid bolus, antibiotics, and pressor support (norepinephrine), steroids	emergent right heart catheterization, placement of a percutaneous transvalvular left ventricular support device (Impella CP), veno-arterial extracorporeal membrane oxygenation (ECMO), hemodialysis
Hydrocortisone 50 mg, 6-hourly, and broad-spectrum antibiotics (piperacillin/tazobactam and vancomycin). Treatment with intravenous immunoglobulin (3 days), methylprednisolone (3 days), and tocilizumab.	Urgent pericardiocentesis, continuous renal replacement therapy. Coronary angiogram and endomyocardial biopsy performed via right common femoral artery (CFA). Pericardial tamponade requiring insertion of pericardial drain.
no information	an ICD was implanted on the 20th day of admission (due to the risk of sudden cardiac death), Synchronized cardioversion shock at 150 joules was applied with a resolution of the VT to 80 beats per minute of sinus rhythm on ECG re-evaluation
inotropic support with dobutamine (5 µg/kg/min)	Percutaneous subxiphoid pericardiocentesis (evacuation of 200 ml of citrine yellow effusion). Then - The patient was sedated, intubated and mechanical ventilation was instituted. Then - implantation of peripheral VA-ECMO as a bridge to recovery. Then - ventilator-associated pneumonia (Streptococcus pneumoniae and Haemophilus influenzae).

hydroxychloroquine and ceftriaxone (patient allergic to macrolides), adrenaline, levofloxacin (500 mg twice daily), antiviral therapy with remdesivir (100 mg once daily) and oseltamivir (150 mg once daily), anticoagulation therapy with low molecular weight heparin (LMWH) (6,000 IU twice daily), diuretic, pantoprazole (20 mg twice daily) and dexamethasone (10 mg once daily), aripiprazole (20 mg once daily), low doses of ramipril (2.5 mg) and bisoprolol (2.5 mg)	oxygen therapy at 10 L/min with non-rebreather mask,high-flow and then intubation, hemotransfusion,
loading dose of aspirin and fondaparinux, dobutamine, entacavir, antilymphocyte serum, high-dose corticosteroids (CS), and mycophenolate mofetil; maintenance used decreasing dose of CS, mycophenolate mofetil, and cyclosporine.	Emergency pericardiocentesis with removal of 300 mL of serosanguinous exudative fluid, clinical and biological status worsened rapidly progressive biventricular hypokinetic non-dilated cardiomyopathy, intubation, VA-ECMO, inta aortic ballon, heart transplantation

intravenous (IV) fluid boluses, vasopressors, and IV antibiotics, hydroxychloroquine and azithromycin, noradrenaline and vasopressin, tocilizumab, methylprednisolone, and IV immunoglobulin/ aspirin 800 mg three times a day and colchicine 0.5 mg twice a day	intubation/ NR
colchicine (500 µg twice daily) and ibuprofen (400 mg three times daily), 1200 mg per day of ibuprofen, 30 mg per day of prednisolone, codeine phosphate, 0.9% sodium chloride	NR
noradrenalin, dobutamine	intubation, VA-ECMO
methylprednisolone (200 mg/day), and intravenous human immunoglobulin (IVIG; 20 g/day) for 4 days, hydroxychloroquine (800 mg on day 1, then 400 mg/day) and azithromycin (500 mg/day), both during 5 days without a QTc greater than 450 ms, and lopinavir/ritonavir (800/200 mg/day, 10 days), norepinephrine, dobutamine, levosimendan, furosemide	oxygen therapy without mechanical ventilation

intravenous ceftriaxone (1 g once daily), intravenous azithromycin (500 mg once daily), and a loading dose of oral hydroxychloroquine (400 mg twice daily). In addition to antimicrobial therapy, the patient also received intravenous furosemide (60 mg once) for clinical hypervolemia, heart failure treatment	5L/min of oxygen via nasal cannula, then 15L/min, BiPAP
methylprednisolone 100 mg then methylprednisolone 100 mg and tocilizumab 800 mg and tocilizumab 800 mg, high-dose vasopressor support	VA-ECMO, continuous venovenous haemofiltration, biventricular support with a ProtekDuo and axillary Impella 5.0
Serum therapy and intravenous (IV) antibiotic treatment with meropenem 1 g/8 h plus metronidazole 500 mg/8 h, hydroxychloroquine, azithromycin and 600 mg of IV tocilizumab, dobutamine, nitroprusside	intubation

norepinephrine, insulin drip for the management of DKA and broad-spectrum antibiotics coverage with IV vancomycin and piperacillin-tazobactam, potassium, dobuamine, norepinephrine, heparine, methylprednisolone 1 gram daily for three days	intubation, Impella
A: ibuprofen 400mg 3x/d for three weeks<br>acetylsalicyl acid 100mg/ d<br>pantoprazole 40mg/d B:sacubitril/<br>valsartan 24/26mg 2x/d<br>bisoprolol 5mg/d<br>ivabradine 5mg 2x/d<br>eplerenone 25mg/ d<br>torasemid 20mg 2x/d<br>acetylsalicyl acid 100mg/ d<br>clopidogrel 75mg/ d<br>atorvastatin 40mg/d<br>pantoprazole 40mg/ d	NR
1. intravenous fluid resuscitation and a low rate of noradrenaline (0.01 µg/kg/min)<br>2. levosimendan infusion<br>3. high-dose intravenous immunoglobulin (IVIG), dexamethasone and IL-1-blockade (anakinra)	percutaneous ventricular assist device - Impella<br>invasive mechanical ventilation<br>V-A ECMO

Hydroxychloroquine, azithromycin, ceftriax-one, tigecycline, favipiravir, and colchicine	no
colchicine 0.5 mg twice daily, prednisone 40 mg once daily	no
5 mg of ramipril and 5 mg of bisoprolol	no
hydroxychloroquine, azithromycin, and ceftriaxone methylprednisolone, 500 mg intravenously daily for 4 days, with subsequent prednisone taper; colchicine, 0.6 mg every 12 h milrinone, norepinephrine, interleukin-6 inhibitor tocilizumab, aldose reductase inhibitor (ARI) currently known as AT-001	endotracheal intubation and mechanical ventilation neuromuscular blockade with continuous rocuronium infusion
fluid overloading and norepinephrine immunoglobulins (80 mg/d) for 4 days and methylprednisolone (500 mg/d) at tapering doses for 14 days and antiviral treatment consisting of IFN B (0.25 mg/48 h) and ritonavir/lopinavir (400 mg/100 mg/12 h)	balloon counterpulsation and venoarterial extracorporeal membrane oxygenation (ECMO) via the femoral artery, resuscitation, pericardiocentesis

ramipril, bisoprolol and prednisone (1 mg/Kg daily) in addition to anti-viral therapy with remdesivir	no
bisoprolol and lisinopril	no
500-mg dose of azithromycin and a single 75-mg dose of oseltamivir.	NR
Hydroxychloroquine, Intravenous Dexamethasone, Intravenous Immunoglobulin, intravenous Ascorbic acid nd oral Melatonin, broad-spectrum antibiotics, vasopressor and amiodaron, , Midodrine	NR
intravenous diuretics, rate-control agents, anticoagulation, ACE inhibitor and mineralocorticoid antagonist	NR

aspirin 300 mg, clopidogrel300 mg and simvastatin 20mg, proton pump inibitor PPI 40mg, plavix kardegic, converting enzyme inhibitor, bisoprolol, and sars cov 2 infection treatment: vitamin c, zinc, azythromicin, dexamethasone and curative anticoagulation Enoxaparin	hight concentration mask 8l/min
cefotaxime and clindamycin, acetylsalicylic acid 300 mg, furosemide and norepinephrine	(2 L/min) by nasal cannula
Aspirin (3,000 mg/day) and colchicine (1 mg/day), prednisolone	NR
subcutaneous anakinra (100 mg daily), an interleukin-1 receptor antagonist	NR

moxicillin with clavulanic acid, clarithromycin, and antipyretics	bone marrow and lymph node biopsy
aspirin (81 mg once daily), Clopidogrel (75 mg once daily) and colchicine (0.6 mg twice daily), Enalapril (2.5 mg once daily) and carvedilol (3.125 mg twice daily)	NR
500 mg oral azithromycin, 1 g ceftriaxone i.v/daily. 125 mg i.v methylprednisolone/ 1x, 40 mg prednisone p.o / daily, aspirin 325 mg, ticagrelor 180 mg, and a heparin infusion (initial bolus 60 U/kg, followed by 12 U/kg/h). dobutamine, norepinephrine, furosemide, vancomycin and piperacillin/tazobactam	Central venous catheter was placed and showed low cardiac output with a significantly reduced mixed venous O2 saturation of 28%

norepinephrine , heparin, tocilizumab, hydroxychloroquine, oseltamivir, azithromicin	veno-arterial extra- corporeal membrane oxygenation (VA-ECMO), continuous venous-venous haemodiafiltration
Vasopressors, Inotropes, Azithromycin, Vancomycin, Cefepime, IVIG	Pericardiocentesis
angiotensin-converting enzyme inhibitors and beta-blockers, anticoagulant and anti-arrhythmic therapy, antibiotic treatment	NR
cyclophosphamide and high-dose steroid	no
Hydroxychloroquine and azithromycin, broad-spectrum antibiotics and hydrocortisone	ECMO
cetaminophen intravenous (IV) drip, ceftriaxone, and vancomycin, hydroxychloroquine and azithromycin, dobutamine, indomethacin, Intravenous methylprednisolone and colchicine	no

acetaminophen	no
hydroxychloroquine, azithromycin, bisoprolol, tocilizumab, methylprednisolone	NR
adrenaline, noradrenaline	non-invasive ventilation, IABP, VA-ECMO, ortotra

Complications (cardiogenic shock, ARDS, heart failure, multi-organ failure etc., with day of occurance)	Outcome (death/recovered/undetermined) with time
ARDS	NR
NR	NR
NR	NR
NR	recovered, discharged on day 13
ARDS, mixed distributive and cardiogenic schock	recovered, discharged on day 12

Cardiogenic schock	recovered, transferred to the previous for rehabilitation hospital on the 24th day after adission
NR	NR
heart failure	recovered, duration of hospitalization NR
heart failure	At the time of submission, the patient still hospitalized with progressive clinical and hemodynamic improvement.
cardiogenic shock and pulmonary infection	Reported that the myocardial injury markers fully recovered to the normal range after 3 weeks of treatment.
septic shock and DIC	death on the 33rd day of hospitalization

cardiogenic tamponade with concurent cardiogenic shock	discharged home 28 days after admission
cardiogenic and vasodilatory shock	discharged home 23 days after admission on intermittent hemodialysis, which was stopped 3 weeks later after improvement of the renal function
cardiogenic and vasodilatory schock	recovery. After 14 days of hospitalization, the patient was discharged home. At this time, both MSC devices were explanted.
NR	death. she died due to cardiac arrest (less than 24 h after admission)
NR	recovery. discharged after 2 weeks with an ejection fraction of 45%, Follow-up of the patient for 3 months revealed an LVEF of 45% and normal RV function.

NR	recovery. Follow-up of the patient for 3 months showed an LVEF of 45%–50%
NR	recovery. discharged after 7 days with stable hemodynamics. Follow-up of the patient for 3 months showed an LVEF of 45% and a normal cTnI value.
NR	recovery. discharged in a satisfying condition. Follow-up of the patient for 3 months showed an LVEF of 45%, mild pericardial effusion, and normal right ventricular size and function.
NR	recovered within 3 days and was discharged in a satisfying condition. Follow-up of the patient for 3 months revealed LVEF of 45%–50% and a normal cardiac function
NR	recovery, discharged in a satisfying condition after 10 days. Follow-up of the patient over 3 months demonstrated an LVEF of 50%

NR	recovery. discharged in a satisfying condition
	the patient died of cardio-respiratory failure within 48 h of admission
NR	recovery (10 DOHS)
-	RECOVERY - The patient was ultimately discharged for physical rehabilitation on hospital day 52.
Cardiogenic schock	death on day 5 due to intractable cardiogenic shock

combined right and left ventricular failure, multiorgan failure	significant improvement in hemodynamics and ejection fraction over the next 5 days
Pulseless electrical activity cardiac arrest with extra-corporeal membrane oxygenation (ECMO) and cardiopulmonary resuscitation (CPR). Distal perfusion cannulae placed. Development of ischaemic right lower leg (without ECMO cannulae) requiring DSA and fasciotomy.	death, after 9 days of hospitalization
none	Recovery. The patient remained asymptomatic for the rest of the hospitalization period and was discharged home after 22 days of hospitalization
Cardiogenic shock in less than 12 h with signs of systemic hypoperfusion (systemic arterial pressure <60 mm Hg, sweating, vomiting, oliguria, and lactate 4.5 mmol/L) and further degradation of the left ventricular systolic function (LVEF 10%, left ventricular outflow tract time-velocity integral 6 cm) despite an escalation of the inotropic (dobutamine 10 µg/kg/min) and vasopressor (noradrenaline 0.17 µg/kg/ min) support.	Recovery. At 3-month follow-up, echocardiography confirmed the complete myocardial recovery (LVEF 65%) and cardiac magnetic resonance imaging showed no signs of myocardial residual fibrosis.

clinical deterioration due to cardiogenic shock with severe hypotension, tachycardia, oliguria, anemia, and marked desaturation	Recovery. After 3 weeks, the patient was discharged, hemodynamically stable and asymptomatic.
patient became hypotensive and hypoxaemic (nasal cannula, oxygen 4 L/min) with clinical signs of pulmonary oedema, rapidly progressive biventricular hypokinetic non-dilated cardiomyopathy, myocarditis fulminant with cardiogenic shock	The patient fully recovered and was discharged from the hospital for cardiovascular rehabilitation without any persistent respiratory or cardiac symptom.

acute kidney injury, ARDS/ NR	discharged on day 16 /chest pain-free upon discharge
lower limb myositis	discharged day 13
AKI KDIGO 3, need for vasoactive support, cardiogenic shock	deceased 6 days after admission
cardiogenic shock	discharged day 16

respiratory distress	discharged
respiratory distress, hypotensive, rapid atrial fibrillation, respiratory failure, oliguric kidney failure	recovered from critical illness and is currently on the general medical floor awaiting discharge to a rehabilitation centre
cardiogenic shock	One month after discharge, he was completely recovered

ARDS, ventricular tachycardia, cardiogenic shock due to myocarditis fulminant, acute oliguric renal failure	The Impella device was then removed, dobutamine was weaned off, and the patient was extubated the next day.
NR	Discharged
septic shock, severe end-organ failure	recovery

no	recovery
no	recovery
no	recovery
cardiogenic shock, severe ARDS,	recovery
cardiac arrest - electrical activity with no pulse and requiring cardiopulmonary resuscitation, emergent pericardiocentesis (drainage of serous fluid), and high-dose vasopressors for hemodynamic recovery	recovery

no	recovery
no	NR
NR	NR
a combination of cardiogenic and septic shoc, respiratory arre	death
NR	recovery

NR	discharged on day 7
respiratory distress with oliguria and hypotension, facial nerve palsy after discharge	discharged 11 days after admission
Ten days after admission, the patient also had bilateral peripheral facial nerve<br>palsy; he could not close his mouth completely and had a drooping corner of the mouth that was more severe on the<br>left side than on the right. He was also unable to winkle his<br>forehead, and brain magnetic resonance imaging showed no<br>abnormal findings	discharged 24 days after admission
NR	recovered

Kikuchi-Fujimoto disease	dsicharged, at 7 months physical impairment persists, and on echocardiography reduced ejection fraction (53%) with impaired global systolic heart function is still present
no	discharged
with acute on chronic respiratory failure	death

cardiogenic shock secondary to severe myocarditis	recovery. The patient's condition improved gradually, off oxy- gen, and a repeat transthoracic echocardiogram at 60 days showed improved cardiac function with a left ven- tricular ejection fraction of 50%.
gastro-intestinal bleeding and multi-organ failure.	death (4 DOHS)
NR	recovery, day 9
acute renal injury with microhaematuria	recovery
cardiogenic shock, ARDS	death (7DOHS)
ARDS	NR

no	NR
no	recovery
cardiogenic shock, sepsis	death
